# EFL-3/E2F7 modulates Wnt signalling by repressing the Nemo-like kinase LIT-1 during asymmetric epidermal cell division in *Caenorhabditis elegans*

**DOI:** 10.1242/dev.204546

**Published:** 2025-03-03

**Authors:** Mar Ferrando-Marco, Michalis Barkoulas

**Affiliations:** Department of Life Sciences, Imperial College, London SW7 2AZ, UK

**Keywords:** *C. elegans*, E2F family, EFL-3/E2F7, Asymmetric division, Seam cell, Wnt signalling, LIT-1/NLK

## Abstract

The E2F family of transcription factors is conserved in higher eukaryotes and plays pivotal roles in controlling gene expression during the cell cycle. Most canonical E2Fs associate with members of the Dimerisation Partner (DP) family to activate or repress target genes. However, atypical repressors, such as E2F7 and E2F8, lack DP interaction domains and their functions are less understood. We report here that EFL-3, the E2F7 homologue of *Caenorhabditis elegans*, regulates epidermal stem cell differentiation. We show that phenotypic defects in *efl-3* mutants depend on the Nemo-like kinase LIT-1*.* EFL-3 represses *lit-1* expression through direct binding to a *lit-1* intronic element. Increased LIT-1 expression in *efl-3* mutants reduces POP-1/TCF nuclear distribution, and consequently alters Wnt pathway activation. Our findings provide a mechanistic link between an atypical E2F family member and NLK during *C. elegans* asymmetric cell division, which may be conserved in other animals.

## Teaser

**Summary:** A combination of functional genetics, NanoDam and quantitative imaging reveals a role for an atypical E2F, EFL-3/E2F7, in epidermal stem cell fate determination in *C. elegans*.

## INTRODUCTION

Development of multicellular organisms depends on the formation of different tissues and specialised cell types. Stem cells are central in this process having the potential to give rise to cellular diversity through asymmetric cell division, while they are also maintained in an undifferentiated state and can increase their population through symmetric division (Slack, 2018). The balance between symmetric and asymmetric divisions needs to be tightly regulated because defects in stem cell regulation can compromise tissue homeostasis and increase the risk of cancer (Bajaj et al., 2020; Morrison and Kimble, 2006). Therefore, studying key genes and pathways regulating stem cell behaviour is central for unravelling mechanisms at play in healthy and disease states.

**Fig. 1.**
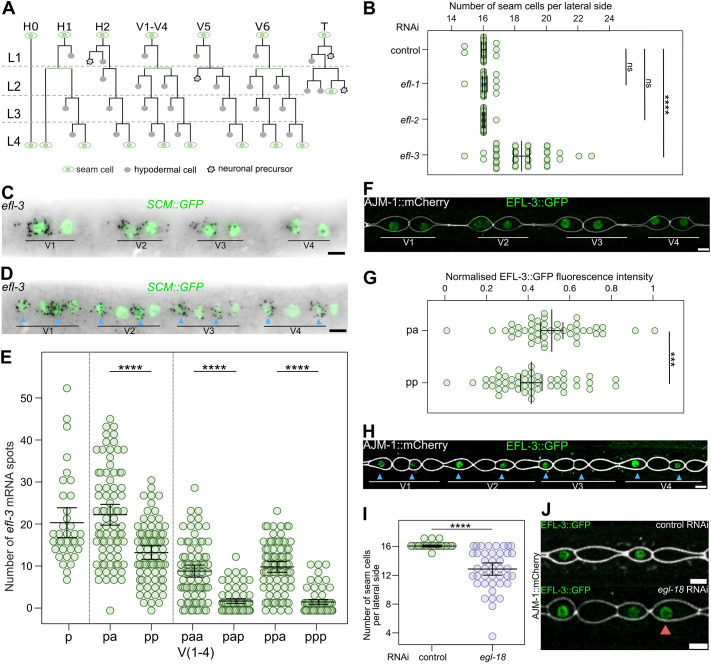
**EFL-3 is an E2F transcription factor regulating seam cell development.** (A) Schematic showing seam cell divisions from L1 to L4. Seam cells are labelled in green, hypodermal cells in grey and neuroblasts in purple. (B) Seam cell number counts upon RNAi targeting members of the E2F family in *C. elegans*. Unlike *efl-1* and *efl-2*, downregulation of *efl-3* leads to a significant increase in the terminal seam cell number (*P*<0.001 with a two-tailed *t*-test, *n*=30 animals per condition). ns, not significant. (C,D) Representative *efl-3* smFISH images following the L2 symmetric division (C), and the L2 asymmetric division (D). Seam cell nuclei are labelled using *SCM::GFP*. (E) Quantification of *efl-3* mRNA spots in V1-V4 seam cells before (p) and after the L2 symmetric division (pa, pp), and following the L2 asymmetric division (paa, pap, ppa, ppp); *n*=36 (p), *n*=78 (pa, pp), *n*=83 (paa, pap, ppa, ppp) animals per condition. (F) Representative image of EFL-3::GFP before the L2 asymmetric division. (G) Quantification of EFL-3::GFP fluorescence intensity before the L2 asymmetric division in V1-V4 lineages. Anterior cells show higher amounts of EFL-3 compared to posterior cells (*P*<0.01 with a two-tailed *t*-test, *n*=45 cells per condition). (H) Representative image of EFL-3::GFP after the L2 asymmetric division. (I) *egl-18* RNAi-treated animals show a statistically significant decrease in the mean seam cell number compared to control animals (*P*<0.001 with a two-tailed *t*-test, *n*=41 animals per condition). (J) Posterior daughter cells (example marked with red arrowhead) show expression of EFL-3::GFP when treated with *egl-18* RNAi following the asymmetric L3 division; *n*=91 cells per condition. Blue arrowheads in D and H point to anterior daughter cells. In F, H and J the membrane of the seam cells is labelled using the apical junction marker *ajm-1p::ajm-1::mCherry*. Error bars in B, E, G and I show mean±s.d. Scale bars: 5 µm (C,D,F,H,J). See also Fig. S1.

The epidermal seam cells of *Caenorhabditis elegans* serve as a model to study stem cell regulation (Joshi et al., 2010). Seam cells are linearly arranged throughout the length of the body (named H0-H2, V1-V6, and T) and divide symmetrically and asymmetrically in a stem-like manner during postembryonic development (Fig. 1A). Following most seam cell asymmetric divisions, posterior daughter cells maintain the seam cell fate, whereas anterior daughter cells endoreduplicate and fuse to the hyp7 epidermal syncytium (Hedgecock and White, 1985; Sulston and Horvitz, 1977). The seam cells generate the majority of the epidermal nuclei and thus the cuticle, which is essential for growth and protection from biotic and abiotic stress (Chisholm and Hsiao, 2012). The H2, V5 and T seam cell lineages also give rise to neuronal precursors (Sulston and Horvitz, 1977). The seam cells expand their population from 10 to 16 through a symmetric division at the early L2 stage, when both daughter cells maintain proliferative potential. At the end of larval development, the 16 seam cells terminally differentiate and fuse into another syncytium that generates the cuticle ridges called alae (Altun and Hall, 2002). Seam cell divisions and their position along the anterior-posterior axis are highly invariant, allowing the characterisation of phenotypic errors at single-cell resolution (Katsanos et al., 2017).

Seam cell development is controlled by Wnt signalling, a widely conserved pathway consisting of intercellular signals that can travel in the body to shape tissues by influencing cell polarity and proliferation (Gleason and Eisenmann, 2010; Pani and Goldstein, 2018; Yamamoto et al., 2011). Correct seam cell patterning relies on the Wnt/β-catenin asymmetry pathway (WβA). This pathway ensures that, following asymmetric division, posterior daughter cells activate Wnt targets and retain seam cell fate, while anterior daughter cells do not activate Wnt and instead differentiate (Jackson and Eisenmann, 2012; Sawa and Korswagen, 2013). In the dividing mother cell, Wnt ligands drive positive regulators, such as Frizzled receptors and Dishevelled, to localise to the posterior cortex (Goldstein et al., 2006; Green et al., 2008; Mizumoto and Sawa, 2007b). Instead, negative WβA regulators, such as APR-1/APC and PRY-1/Axin asymmetrically localise to the anterior cortex (Baldwin and Phillips, 2014; Mizumoto and Sawa, 2007a). The β-catenin WRM-1 also localises to the anterior cortex and recruits LIT-1/NLK and more APR-1/APC and PRY-1/Axin (Mizumoto and Sawa, 2007a; Takeshita and Sawa, 2005). In turn, APR-1 promotes anterior nuclear export of WRM-1 through regulation of microtubule dynamics, while absence of APR-1 from the posterior daughter cell after division leads to nuclear WRM-1 localisation (Mizumoto and Sawa, 2007b; Sugioka et al., 2011). Moreover, cortical APR-1 promotes SYS-1/β-catenin degradation through the destruction complex, leading to asymmetric localisation of SYS-1 to the posterior nucleus (Baldwin and Phillips, 2014). In posterior daughter cells, high nuclear WRM-1 binds to LIT-1, promoting its activation by MOM-4/MAPK and nuclear translocation (Shin et al., 1999; Takeshita and Sawa, 2005; Yang et al., 2015). Nuclear LIT-1 phosphorylates POP-1, the sole Tcf/Lef homologue of *C. elegans*, leading to its nuclear export (Lo et al., 2004; Meneghini et al., 1999; Rocheleau et al., 1999; Yang et al., 2011). Low POP-1 and high SYS-1 levels in posterior daughter nuclei allow the formation of a TCF/β-catenin transcriptional activator complex (POP-1/SYS-1) that activates Wnt targets (Huang et al., 2007; Jackson and Eisenmann, 2012; Phillips et al., 2007; Shetty et al., 2005), such as the GATA transcription factor EGL-18 (Gorrepati et al., 2013). In anterior daughter cells, high POP-1 and low SYS-1 levels in the nucleus lead to recruitment of co-repressors and transcriptional repression of Wnt targets (Bekas and Phillips, 2022 preprint; Phillips et al., 2007). Besides Wnt signalling, seam cell patterning is also regulated by other conserved transcription factors, such as a Runx/CBFβ module, CEH-16/engrailed and various GATA factors, which can influence the decision between stem cell fate maintenance or differentiation (Huang et al., 2009; Kagoshima et al., 2007; Katsanos and Barkoulas, 2022; Koh and Rothman, 2001; Nimmo and Woollard, 2008; Smith et al., 2005; Xia et al., 2007). The timing of seam cell divisions is tightly controlled by the heterochronic genes, which include *lin-4* and *let-7* microRNA families, regulating the abundance of specific targets that are necessary for developmental progression (Ambros and Horvitz, 1984; Arasu et al., 1991; Lee et al., 1993; Reinhart et al., 2000; Rougvie and Moss, 2013; Wightman et al., 1993).

The E2F family of transcription factors (E2F1-8 in humans) controls gene expression during the cell cycle. E2F1-6 associate with DP proteins to form heterodimers that control the expression of cell cycle genes (Attwooll et al., 2004; Van Den Heuvel and Dyson, 2008). Atypical E2F7-8 lack a DP-binding domain and are thought to act as transcriptional repressors (Christensen et al., 2005; De Bruin et al., 2003; Di Stefano et al., 2003; Lammens et al., 2009; Logan et al., 2005). Most E2Fs are regulated during the cell cycle through direct binding to pocket proteins, such as the tumour suppressor RB. These interactions are in turn regulated by the activity of cyclin-dependent kinase (CDK) and cyclin complexes, which phosphorylate pocket proteins causing E2F release (Attwooll et al., 2004). The CDK-RB-E2F pathway is essential to prevent abnormal proliferation and tumour growth, with unrestrained E2F-mediated transcription being a key driver for many cancers (Kent and Leone, 2019; Xie et al., 2021). While E2Fs have been largely studied in the context of cell division, it is known that they also have non-canonical functions beyond the cell cycle (Cam and Dynlancht, 2003; Zalmas et al., 2008). The *C. elegans* genome contains three homologues of the mammalian E2F family, *efl-1*, *efl-2* and *efl-3*. EFL-1 and EFL-2 bind to DP/DPL-1 to repress expression of genes ensuring correct development of tissues such as the vulva (Chen and Han, 2001; Page et al., 2001; Van Den Heuvel and Dyson, 2008). For example, *efl-1* is part of the DRM complex and the SynMuvB gene regulatory pathway, which inhibits vulval development and prevents somatic cells from acquiring a germline fate (Ceol and Horvitz, 2001; Kudron et al., 2013). In addition, *efl-1* and *efl-2* have been described to have pro-apoptotic function in the germline (Schertel and Conradt, 2007). Previous research on *efl-3*, the E2F7 homologue in *C. elegans*, has suggested functions in preventing apoptosis of ventral cord neurons and regulation of cell numbers in the somatic gonad and epidermis (Winn et al., 2011; Katsanos et al., 2021; Soukup et al., 2022).

In this study, we characterise in detail the role of EFL-3 in seam cell development. We report that EFL-3 localises preferentially in anterior daughter cells following asymmetric seam cell division and this localisation depends on Wnt signalling. Tissue-specific *efl-3* mutants display gains and losses of seam cells. These phenotypes are dependent on the Nemo-like kinase gene *lit-1*, which we show to be a direct EFL-3 target. Increased *lit-1* expression reduces POP-1 levels in the seam cell daughter nuclei leading to changes in seam cell fate specification. Our study therefore provides evidence that the atypical E2F EFL-3 modulates epidermal stem cell patterning in *C. elegans* through regulation of *lit-1* expression, and, consequently, Wnt signalling pathway activity.

## RESULTS

### EFL-3 shows an asymmetric distribution following seam cell division

As part of our efforts to gain insights into the machinery regulating seam cell development, we previously performed transcriptional profiling and identified *efl-3* as a seam cell-expressed transcription factor causing seam cell hyperplasia when downregulated (Katsanos et al., 2021). The *C. elegans* genome contains three E2F family members, *efl-1*, *efl-2* and *efl-3*, but only downregulation of *efl-3* led to a significant change in the average number of seam cells based on expression of the *SCM::GFP* marker (Koh and Rothman, 2001) (Fig. 1B).

To characterise the *efl-3* expression pattern in seam cells, we used single-molecule fluorescence *in situ* hybridisation (smFISH). We focused on the V1-V4 lineages, which exhibit similar division patterns, and the L2 larval stage, during which seam cells divide twice, first symmetrically and then asymmetrically. Interestingly, we observed an asymmetric localisation of *efl-3* transcripts between the two daughter cells following the symmetric and asymmetric L2 divisions (Fig. 1C,D). In both cases, anterior daughter cells displayed a higher number of *efl-3* mRNA transcripts compared to posterior daughters (Fig. 1E). Following the L2 asymmetric cell division, posterior daughter cells presented a strong reduction in *efl-3* transcript abundance, with 50% of cells showing no *efl-3* mRNAs at all (Fig. 1E). There was no evidence for asymmetric inheritance of *efl-3* mRNAs (Fig. S1A-D). Therefore, *efl-3* mRNA asymmetry may arise from differences in *efl-3* transcription or changes in the stability of *efl-3* transcripts between anterior and posterior cells.

To assess whether this asymmetric mRNA distribution also reflected how EFL-3 is distributed at the protein level, we generated and imaged an *efl-3::gfp* knock-in strain. Consistent with the mRNA transcript enrichment in anterior cells, EFL-3::GFP expression was moderately enriched in anterior daughter cells after symmetric division in comparison to posterior daughters (Fig. 1F,G). Following the L2 asymmetric division, this enrichment became more pronounced, with EFL-3::GFP expression specifically observed in anterior daughter cells (Fig. 1H). We hypothesised that the Wnt pathway, which is normally activated in posterior daughter cells retaining the seam cell fate, may contribute to the *efl-3* repression seen in these cells. To test this hypothesis, we knocked down the Wnt downstream effector *egl-18* by RNA interference (RNAi), which results in loss of seam cell fate maintenance and decreased terminal seam cell number (Fig. 1I). Notably, we found EFL-3::GFP to be expressed in 21% of posterior daughter cells in *egl-18* RNAi animals compared to none in the control treatment (*P*<0.005 with a χ^2^ test; *n*≥91 cells per condition) (Fig. 1J). Taken together, these results highlight that EFL-3 distribution in seam cells is asymmetric and that this asymmetry is dependent on Wnt pathway activation.

### Loss of *efl-3* function leads to gains and losses of seam cells during larval development

**Fig. 2.**
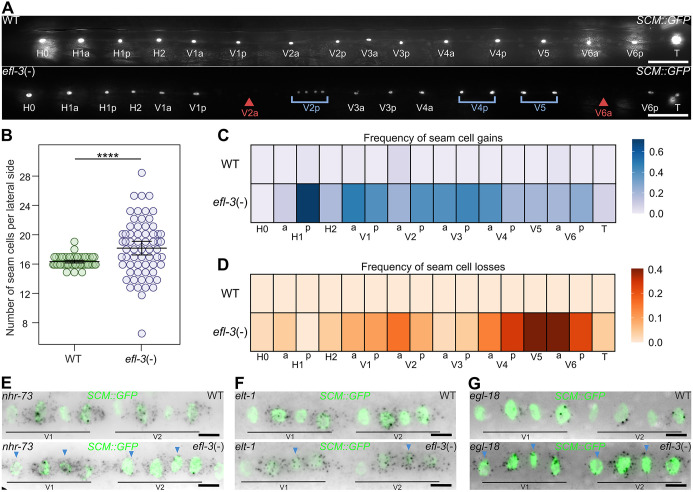
**A tissue-specific *efl-3* mutant displays gains and losses of seam cells.** (A) Representative images of tissue-specific *efl-3* mutant animals [simplified here as *efl-3*(−)] versus floxed animals not expressing the Cre recombinase (WT) at the early adult stage. Blue brackets indicate lineages with seam cell gains; red arrowheads point to lineages with seam cell losses. (B) Seam cell counts in tissue-specific *efl-3* mutant animals at the early adult stage show a significant increase in the average number of seam cells in comparison to controls (*P*<0.001 with a two-tailed *t*-test, *n*≥58 animals per condition). Error bars show mean±s.d. (C,D) Heatmaps showing the frequency of seam cell gains (C) and losses (D) per cell lineage at the end of postembryonic development in tissue-specific *efl-3* mutant animals versus controls (*n*=50 animals per condition) a, anterior lineage; p, posterior lineage. (E-G) Representative smFISH images of *nhr-73* (E), *elt-1* (F) and *egl-18* (G) mRNA distribution in tissue-specific *efl-3* mutant animals in comparison to controls following the L2 asymmetric division. Blue arrowheads point to anterior daughter cells that ectopically express *nhr-73*, *elt-1* and *egl-18* in *efl-3* mutant animals. Seam cell nuclei are labelled using *SCM::GFP*. Scale bars: 50 µm (A); 5 µm (E-G). See also Figs S2 and S3.

To consolidate the RNAi results, we sought to study the impact of strong loss of *efl-3* function on seam cell development. To circumvent the fact that *efl-3* is an essential gene (Winn et al., 2011), we constructed a seam cell-specific mutant by inserting *loxP* recombination sites flanking the *efl-3* locus and introducing this modification into a genetic background expressing Cre recombinase specifically in the seam cells. We confirmed that neither the introduction of *loxP* sites flanking the *efl-3* locus nor the expression of Cre recombinase in the seam cells disrupt seam cell development (Fig. S2A). Furthermore, transgenic *efl-3^loxP^* animals carrying the Cre recombinase did not show any *efl-3* expression in the seam cells by smFISH, hence they are likely to represent seam cell-specific null mutants (Fig. S2B). We found that tissue-specific *efl-3* mutants displayed a stronger phenotype than *efl-3* RNAi-treated animals, with both gains and losses of seam cells observed at the early adult stage (Fig. 2A,B). Gains of seam cells were more frequent in the H1p and anterior V lineages (Fig. 2C), whereas seam cell losses were more frequently observed in the posterior V lineages (Fig. 2D). When *efl-3* mutants were treated with *egl-18* RNAi, the seam cell loss phenotype was enhanced (Fig. S2C). Taken together, these results suggest that EFL-3 can both promote and repress seam cell fate.

To determine at what larval stage seam cells are gained or lost in the *efl-3* mutant, we carried out detailed lineage analysis during post-embryonic development. We found that at the late L2 and late L3 stages, *efl-3* mutant animals only displayed seam cell gains (Fig. S2D-F). Gaps in the seam cell line corresponding to seam cell losses were only observed at the late L4 stage, as shown by the loss of expression of the seam cell marker *wrt-2p::GFP::PH* (Fig. S2G) and the nuclear hormone receptor *nhr-73* by smFISH (Fig. S2H). Therefore, lineage analysis suggests that seam cell losses in *efl-3* mutants occur following the L4 asymmetric division and thus succeed the onset of gains.

To characterise the developmental state of the anterior V1-V4 cells that retain the expression of *SCM::GFP* following division, we investigated the expression pattern of other transcription factors whose expression is known to correlate with seam cell fate, such as *nhr-73*, *elt-1* and *egl-18* (Cao et al., 2017; Gorrepati et al., 2013; Miyabayashi et al., 1999; Smith et al., 2005)*.* In all cases, we found that *efl-3* mutants ectopically expressed these genes in anterior daughter cells following asymmetric division (Fig. 2E-G, Fig. S3A-C). In the case of *egl-18*, we confirmed this result at the protein level by studying the expression of *egl-18* endogenously tagged with *mNeonGreen* (Ma et al., 2021). We found a significant increase in EGL-18 protein expression in anterior daughter cells in *efl-3* mutants compared to control (Fig. S3D,E). These results suggest that the gains of seam cells in *efl-3* mutants are driven by anterior daughters failing to differentiate and retaining the seam cell fate following asymmetric division.

Because E2Fs, including E2F7, are known to participate in the regulation of the cell cycle (Chen et al., 2012; Pandit et al., 2012), we examined whether the defects in cell differentiation in *efl-3* mutant animals may be an indirect consequence of cell cycle perturbation. To compare seam cell division progression between wild-type and *efl-3* mutant animals, we quantified the division state of V1-V4 lineages at the L2 stage, when *efl-3* mutants exhibit patterning defects. We observed no significant differences between wild type and *efl-3* mutants in the proportion of lineages undergoing versus completing the L2 asymmetric division (Fig. S4A). Additionally, division asynchrony within each animal showed no notable differences between wild type and *efl-3* mutants (Fig. S4B). Furthermore, following the L2 asymmetric division, wild-type anterior daughter cells endoreduplicate before fusing to the hyp7 syncytium (Altun and Hall, 2002). We reasoned that if anterior cells fail to differentiate in *efl-3* mutants due to a cell cycle defect, we might observe a change in anterior versus posterior cell DAPI staining between the wild type and *efl-3* mutants. However, we found that *efl-3* mutants showed a similar pattern to wild type (Fig. S4C,D). Taken together, these findings suggest that the observed phenotypes in *efl-3* mutants are less likely to result from gross changes in the seam cell cycle, and thus may be associated with changes in cell differentiation.

### Identification of putative EFL-3 targets via NanoDam

To identify downstream targets of EFL-3 in the seam cells, we used a modified Targeted DamID (TaDa) approach for streamlined tissue-specific transcription factor binding called NanoDam (Tang et al., 2022; Yee et al., 2023 preprint). NanoDam employs the main TaDa principle to overcome the considerable toxicity observed when Dam-fusions are expressed at high levels in animal cells (Katsanos and Barkoulas, 2022; Southall et al., 2013). Briefly, transgenes are expressed at very low levels in the tissue of interest via a bicistronic mRNA cassette design harbouring two open reading frames (ORFs). The first ORF encodes a fluorescent protein and is followed by two STOP codons and a frameshift mutation before the ATG of the second ORF. Because of the universal property of eukaryotic ribosomes to reinitiate translation after a STOP codon at a reduced frequency, this transgene configuration leads to low levels of Dam-Protein of interest fusion production. NanoDam relies on the tissue-specific expression of a vhhGFP4 nanobody fused to Dam. This allows profiling of putative targets at the desired tissue upon crossing the GFP nanobody:Dam transgene to a TF-GFP transgenic line (here the *efl-3::gfp* knock-in strain). We performed EFL-3 NanoDam at the L4 stage using both C-terminal and N-terminal fusions of the Dam:GFP-nanobody transgene expressed in the seam cells. The functionality of the EFL-3::GFP fusion was assessed by scoring the seam cell number in heterozygous +*srf-3i::cre efl-3^loxP^*/*efl-3::gfp* animals, which showed a wild-type seam cell number similar to heterozygous +*srf-3i::cre efl-3^loxP^/wt* animals (Fig. S5A).

Aggregate genome-wide EFL-3 NanoDam signal profiles across genes containing statistically significant peaks between 5 kb upstream of the transcriptional start site (TSS) and 2 kb downstream of the transcription end site (TES) showed signal enrichment in sequences proximal to the TSS and the TES (Fig. S5B). We identified 1316 and 1237 statistically significant peaks in the case of the C- and N-terminal Dam:GFP-nanobody fusions, respectively (Table S1), with >70% of peak overlap between the two datasets (Fig. S5C). Hierarchical clustering of the localisation and score of those peaks that lie between 5 kb upstream and 2 kb downstream of genes revealed clusters of signal enrichment upstream of the TSS, within the genes and downstream of TES regions (Fig. S5D). Peaks were assigned to the closest gene leading to 1882 and 1826 genes for each configuration with an 80% overlap between the two gene datasets. The list of candidate EFL-3 target genes showed significant overlap with genes expressed in seam cells based on single-cell combinatorial indexing RNA-sequencing data (Cao et al., 2017) and with a filtered list of genes with known roles in seam cell development from the literature (Table S2). Gene ontology (GO) term analysis of putative EFL-3 targets in the seam revealed terms relating to signalling and transcription, but not cell cycle (Fig. S5E,F).

### EFL-3 controls *lit-1* expression in seam cells

**Fig. 3.**
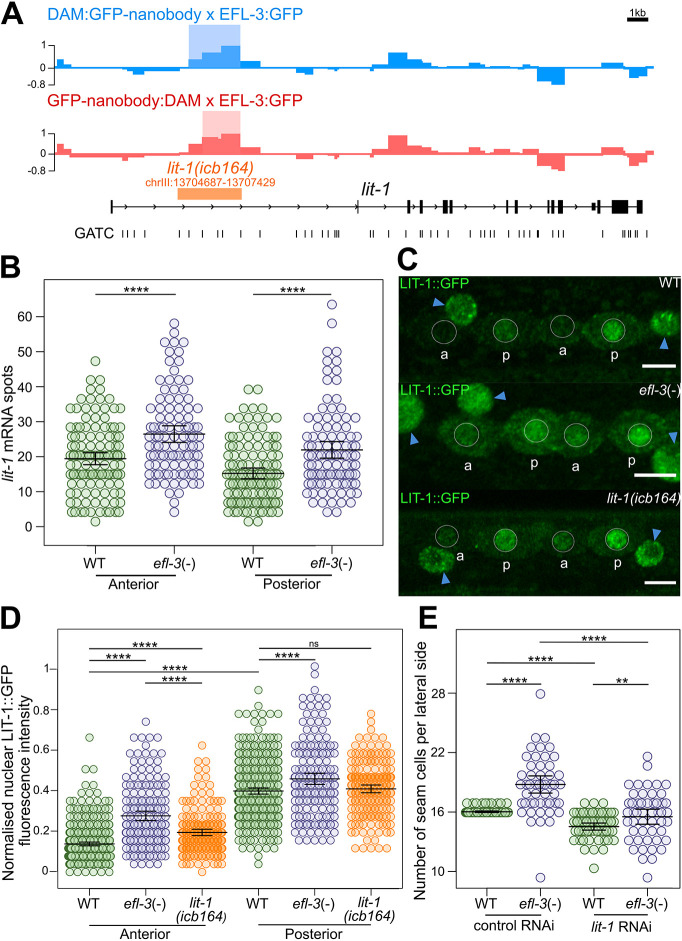
**EFL-3 directly represses *lit-1* expression.** (A) Signal profiles for C-terminal (blue) and N-terminal (red) NanoDam reflecting EFL-3::GFP binding near the *lit-1* locus. The *y*-axis represents log_2_(EFL-3::GFP; GFP-nanobody:dam/GFP-nanobody:dam) score. Statistically significant peaks are indicated by lightly shaded rectangles on the signal tracks (FDR<0.05). The generated *lit-1(icb164)* CRISPR deletion is shown in orange. (B) Quantification of *lit-1* mRNA spots in V1-V4 lineages in anterior and posterior daughter cells in *efl-3* tissue-specific mutants [*efl-3*(−)] versus controls (WT) following the L2 asymmetric division; *n*≥100 cells per condition. (C) Representative images of LIT-1::GFP following the L2 asymmetric division in controls (WT), tissue-specific *efl-3* mutants [*efl-3*(−)] and animals containing the *lit-1(icb164)* CRISPR deletion. Seam cell nuclei are circled in white. Hypodermal nuclei are labelled with blue arrowheads. a, anterior daughter cell; p, posterior daughter cell. (D) Quantification of LIT-1::GFP fluorescence intensity in the nuclei of V1-V4 seam cells following the L2 asymmetric division in controls (WT), tissue-specific *efl-3* mutants [*efl-3*(−)] and animals containing the *lit-1(icb164)* CRISPR deletion; *n*≥175 cells per condition. (E) Seam cell counts in *efl-3* tissue-specific mutant animals versus controls treated with *lit-1* RNAi or empty vector. Error bars in B, D and E show mean±s.d. ****P<0.001, **P<0.01 (two-tailed *t*-test; *n*=50 animals per condition). Scale bars: 1 kb (A); 5 µm (C). See also Figs S5, S6 and S7.

Within the list of putative EFL-3 targets, we focused on candidates with previously reported roles in seam cell development and prioritised them based on smFISH validation of target gene expression changes in an *efl-3* mutant background (Fig. S6A-M). One potential target reproducibly identified in the NanoDam datasets that stood out was *lit-1*, which encodes a MAP kinase homologue of the human NLK and is known to participate in Wnt signal transduction by regulating the nuclear distribution of POP-1 (Lo et al., 2004; Meneghini et al., 1999; Rocheleau et al., 1999; Takeshita and Sawa, 2005; Yang et al., 2011). NanoDam profiling revealed significant signal enrichment in the first intron of *lit-1* (Fig. 3A), in a region that contained a TTTCCGGTCAAA element that resembles the E2F8 binding motif (*P*=1e−5 based on FIMO analysis). We carried out *lit-1* smFISH to compare *lit-1* expression between wild type and *efl-3* mutants after the L2 asymmetric division. We found a significant increase in *lit-1* transcript levels in anterior and posterior daughter cells of *efl-3* mutants, suggesting that EFL-3 may act as a repressor of *lit-1* expression in the seam cells (Fig. 3B). Following an asymmetric seam cell division, LIT-1 has been reported to localise preferentially to the nucleus of posterior daughter cells where it phosphorylates POP-1 to trigger its nuclear export (Takeshita and Sawa, 2005). We therefore focused on LIT-1 nuclear levels during symmetric and asymmetric L2 division using a *lit-1::gfp* knock-in strain. Consistent with the smFISH results above, we found that LIT-1 levels were significantly increased in *efl-3* mutant animals in both anterior and posterior daughter cells following symmetric and asymmetric divisions (Fig. 3C,D, Fig. S7A,B).

To address whether the increase in *lit-1* levels could be causative for the seam cell phenotype observed in *efl-3* mutants, we carried out *lit-1* RNAi in the *efl-3* mutant background. We found a significant reduction in the mean seam cell number of *efl-3* mutant animals upon *lit-1* RNAi treatment compared to the control treatment (Fig. 3E). Lineage analysis revealed that this reduction in seam cell number was driven by a statistically significant suppression of seam cell gains in anterior V lineages in *efl-3* mutants (Fig. S7C). Furthermore, we found a statistically significant reduction in posterior V lineage cell losses in *efl-3* mutants treated with *lit-1* RNAi (Fig. S7D), which was masked when looking at the aggregate seam cell number counts. The suppression of seam cell gains and losses observed in *efl-3* mutants suggests that both phenotypes are LIT-1 dependent.

To test whether EFL-3 could directly control the expression of *lit-1* through the identified site on the first intron, we used CRISPR-mediated genome editing to delete the entire EFL-3 NanoDam peak. Animals containing this intronic deletion showed an increase in nuclear LIT-1 levels in anterior daughter cells following the asymmetric L2 division (Fig. 3C,D). We note that this increase was lesser in magnitude than what is observed in *efl-3* mutants, suggesting that this intronic element may not be the only way in which EFL-3 controls *lit-1* expression in the seam cells. Consistent with this hypothesis, *efl-3* RNAi was found to further increase LIT-1 levels in animals carrying the intronic deletion (Fig. S7E,F). We found no significant difference in nuclear LIT-1 levels between wild type and animals containing the *icb164* deletion when both groups were treated with *efl-3* RNAi (Fig. S7E,F). These results suggest that expression changes observed with the *icb164* deletion are EFL-3 dependent, although EFL-3 is likely to regulate *lit-1* expression through additional regulatory elements or indirectly through other intermediate transcription factors.

### Decrease in nuclear POP-1 levels correlates with seam cell gains and losses in *efl-3* mutants

**Fig. 4.**
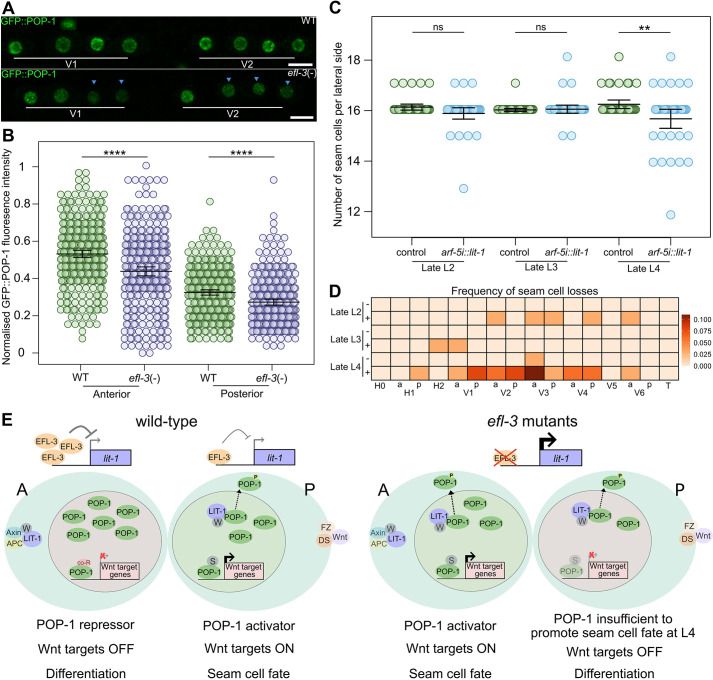
***efl-3* mutants show changes in POP-1 nuclear distribution that are compatible with seam cell gains and losses.** (A) Representative images of GFP::POP-1 following the L2 asymmetric division in tissue-specific *efl-3* mutant animals [*efl-3*(−)] versus controls (WT). Blue arrowheads indicate cells with visually lower expression of GFP::POP-1. Scale bars: 5 µm. (B) Quantification of GFP::POP-1 fluorescence intensity following the L2 asymmetric division in tissue-specific *efl-3* mutant animals versus controls. ****P=3e−9 for anterior daughters and ****P=2e-6 for posterior daughters with a two-tailed *t*-test; *n*≥294 cells per condition. (C) Seam cell counts in wild type (control) and animals overexpressing *lit-1* under the *arf-5i* element (*arf-5i::lit-1*) at the late L2, late L3 and late L4 stages. At late L4, animals overexpressing *lit-1* present a significant decrease in the average number of seam cells compared to the control (**P<0.01 with a two-tailed *t*-test; *n*=36 animals per condition). ns, not significant. (D) Heatmap showing the frequency of seam cell losses per cell lineage at the late L2, late L3 and late L4 stages in wild type (‘−’) and in animals overexpressing *lit-1 (arf-5i::lit-1)* (‘+’). *n*=36 animals per condition, a, anterior lineage; p, posterior lineage. (E) Model of Wnt signalling activation in wild-type and in *efl-3* mutant animals. In wild-type animals, anterior daughters show higher amounts of EFL-3 compared to posterior daughters. Anterior daughters present high POP-1 nuclear levels leading to repression of Wnt target genes and differentiation. Posterior daughter cells exhibit POP-1 nuclear export as a result of LIT-1 and WRM-1 activities. POP-1 interacts with SYS-1 to drive expression of Wnt target genes resulting in seam cell fate maintenance. In *efl-3* mutants, nuclear LIT-1 levels increase in both daughter cells leading to increased POP-1 nuclear export. As a consequence, in L2, L3 and L4 divisions anterior daughter cells show ectopic activation of the Wnt signalling causing gains of seam cells. At L4, POP-1 levels may decrease below the L4-specific threshold and/or independent L4 sensitisation may lead to some posterior daughter cells failing to activate the Wnt signalling and losing seam cell fate. Frizzled and dishevelled localise to the posterior cortex where they bind the Wnt ligand (Wnt). APC (APR-1), Axin (PRY-1), WRM-1 and LIT-1 localise to the anterior cortex. A, anterior daughter cell; co-R, POP-1 co-repressors; DS, dishevelled; FZ, frizzled; P, posterior daughter cell; S, SYS-1; W, WRM-1. Error bars in B and C show mean±s.d. See also Fig. S8.

POP-1 exerts a dual role in seam cell daughters, promoting differentiation as a repressor and the seam cell fate as an activator and these functions are determined by the nuclear POP-1 levels (van der Horst et al., 2019). LIT-1 accumulation in the nucleus of posterior daughter cells is predicted to cause POP-1 nuclear export, thereby ensuring an optimal β-catenin (SYS-1)/POP-1 ratio in the nucleus that is conductive to Wnt target activation (Huang et al., 2007; Phillips et al., 2007; Shetty et al., 2005; Takeshita and Sawa, 2005). Low LIT-1 levels in the nuclei of anterior daughter cells allow POP-1 to also remain at high levels in the nucleus and associate with proteins repressing Wnt target genes (Bekas and Phillips, 2022 preprint; Phillips et al., 2007). We therefore predicted that higher LIT-1 levels in *efl-3* mutant nuclei may have a consequence on POP-1 localisation. To test this prediction, we studied nuclear POP-1 protein levels upon loss of *efl-3* function using a *gfp::pop-1* knock-in strain that is known to be largely functional (van der Horst et al., 2019). In wild-type animals, POP-1 levels were lower in posterior daughter cells in comparison to anterior daughter cells following the L2 asymmetric division (Fig. 4A,B). In *efl-3* mutant animals, we found a significant decrease in POP-1 levels in both anterior and posterior daughter cells (Fig. 4A,B). Changes in POP-1 expression were at the protein level because *pop-1* mRNA transcripts were unchanged between control and *efl-3* RNAi-treated animals (Fig. S8A,B). We also compared POP-1 nuclear levels between the L2 and L4 stage. In wild-type animals, POP-1 expression was higher at the L4 stage compared to L2 (Fig. S8C,D), and a less pronounced asymmetry was observed at the L4 stage between anterior and posterior daughter cells (Fig. S4E). POP-1 levels were reduced to a similar average level in *efl-3* mutants at both the L2 and L4 stages (Fig. S8C,D). These results suggest that while reduction in POP-1 levels can explain the *efl-3* mutant seam cell phenotypes, absolute POP-1 levels alone cannot explain why seam cell losses occur specifically at L4.

To test whether increased *lit-1* expression accounts for the seam cell losses observed in *efl-3* mutants, we overexpressed *lit-1* under the *arf-5i* element, which is contained within the SCM enhancer and is sufficient to drive expression in posterior daughters retaining seam cell fate (Katsanos et al., 2021; Ashley et al., 2021). Animals overexpressing *lit-1* presented losses of seam cells leading to a significant decrease in the mean seam cell number (Fig. 4C). Similar to *efl-3* mutants, losses of seam cells were predominantly observed after the L4 asymmetric division (Fig. 4C,D). As we previously found comparable POP-1 levels in posterior daughter cells of *efl-3* mutants between the L2 and L4 stage, we hypothesised that the losses observed specifically at L4 may reflect an increased sensitivity to change. To test this hypothesis further, we investigated seam cell defects in a strain carrying the hypomorphic *pop-1(hu9)* allele and observed gains of seam cells leading to a significant increase in the mean seam cell number (Fig. S8F). Interestingly, seam cell gains were only observed at the L4 stage (Fig. S8G), suggesting that seam cells are more sensitive to changes in POP-1 activity at this late larval stage.

## DISCUSSION

The E2F family of transcription factors are important for many cellular processes, including cell cycle regulation, apoptosis, and DNA damage (Coutts et al., 2018; Kipreos and van den Heuvel, 2019; Zalmas et al., 2008, 2013). Atypical E2Fs are known to act as transcriptional repressors antagonising canonical E2F activators (Park et al., 2022; Segeren et al., 2020; Thurlings et al., 2017). For example, E2F7 has been implicated in restraining cell proliferation in human cells by directly regulating the expression of genes that regulate mitotic progression among other targets, such as microRNAs (Chen et al., 2012; De Bruin et al., 2003; Mitxelena et al., 2016; Westendorp et al., 2012). Previous work on *efl-3* in *C. elegans* suggested a role in preventing apoptosis in PVA and PVB neurons of the ventral nerve cord by repressing the expression of the pro-apoptotic gene *egl-1* (Winn et al., 2011). In addition, *efl-3* has been implicated in the development of the somatic gonad by specifying the correct amount of distant tip cells (Soukup et al., 2022); however, the exact molecular mechanisms remain to be elucidated. We report here that EFL-3 regulates asymmetric divisions in the *C. elegans* epidermal stem cell model. Consistent with a role as a transcriptional repressor, we show that EFL-3 represses *lit-1* expression in seam cells partially through direct binding to an intronic element, which has a knock-on effect on POP-1 distribution. Our study therefore reveals a link between an atypical E2F repressor and NLK-mediated regulation of Wnt signalling in the context of asymmetric cell division and thus uncovers a role for an atypical E2F beyond their established functions in the cell cycle.

We characterised the *efl-3* expression pattern at the mRNA and protein level and report an enrichment in anterior seam cell daughters after symmetric and asymmetric divisions. This enrichment profile is consistent with a role for EFL-3 in safeguarding cell differentiation of anterior daughter cells. Expression of atypical E2Fs is known to be cell cycle dependent, often peaking at the S phase (De Bruin et al., 2003; Maiti et al., 2005). Following seam cell asymmetric division, anterior daughters immediately proceed to S phase before fusing to the hyp7 syncytium and differentiating, whereas the cell cycle progresses more slowly in posterior daughter cells that will continue to divide (Van Rijnberk et al., 2017). This difference in cell cycle commitment between the two daughter cells could have contributed to the higher *efl-3* expression found in anterior daughter cells after asymmetric division. However, it is unlikely to be the main driver because a similar anterior *efl-3* enrichment was observed following the symmetric division when both daughter cells progress simultaneously to S phase (Van Rijnberk et al., 2017). The asymmetric *efl-3* distribution following symmetric division suggests that the EFL-3 function promoting differentiation can be bypassed at this stage, similar to how Wnt asymmetry is present during symmetric division but also masked at the phenotypic level (van der Horst et al., 2019). We show that downregulation of the Wnt target *egl-18* disrupts the asymmetric expression of EFL-3 following asymmetric division. Therefore, we propose that the anterior enrichment is likely driven by Wnt-dependent repression in posterior daughter cells following an asymmetric cell division. This is reminiscent of the atypical E2F7 repression by Wnt signalling reported in proliferating hepatocytes (Jin et al., 2022). It is of note that, similar to *efl-3* mRNA distribution, *pop-1* transcription was also found to be stronger in anterior daughter cells post-division, which may reflect a negative feedback from Wnt activation on *pop-1* expression. Therefore, besides the known regulation of POP-1 at the protein level (Shin et al., 1999; Yang et al., 2015), transcriptional *pop-1* regulation may further safeguard seam cell fate decisions.

NLK-mediated Tcf/Lef phosphorylation can have positive or negative consequences on its activity depending on the cellular context (Ishitani et al., 1999, 2003; Ota et al., 2012). In *C. elegans*, LIT-1 promotes POP-1 nuclear export allowing low nuclear POP-1 levels in posterior daughter cells and Wnt signalling activation (Lo et al., 2004; Meneghini et al., 1999; Rocheleau et al., 1999; Yang et al., 2011). In *efl-3* mutants, we found higher levels of nuclear LIT-1 and a reduction in nuclear POP-1 levels in both anterior and posterior daughter cells following asymmetric division. LIT-1 activity is regulated by WRM-1 and MOM-4. WRM-1 promotes LIT-1 autophosphorylation, facilitates phosphorylation by MOM-4 and is essential for LIT-1 translocation to the nucleus (Shin et al., 1999; Yang et al., 2015). Although we did not explore interactions with MOM-4 and WRM-1 in this study and cannot rule out that WRM-1 and MOM-4 expression is also altered in *efl-3* mutants, the fact that overexpression of *lit-1* alone is sufficient to reproduce *efl-3* mutant phenotypes suggests that MOM-4 and WRM-1 wild-type activities are likely sufficient to handle the increased amounts of LIT-1 in *efl-3* mutants.

POP-1 acts as either repressor or activator in seam cell daughters depending on its nuclear levels. In anterior cells, high POP-1 levels promote differentiation and a decrease in POP-1 levels has been shown to lead to ectopic Wnt signalling activation and symmetrisation of seam cell divisions (Gleason and Eisenmann, 2010). Posterior daughter cells require low levels of nuclear POP-1, which promotes the seam cell fate. However, if levels of POP-1 drop below a threshold, seam cell fate can no longer be maintained (van der Horst et al., 2019). Therefore, changes in POP-1 nuclear levels through increased *lit-1* expression can contribute to both the seam cell gains and losses observed in *efl-3* mutants (Fig. 4E). In this scenario, lowering POP-1 levels in anterior daughter cells in *efl-3* mutants and upon *efl-3* RNAi can activate Wnt targets and prevent cell differentiation. Meanwhile, lowering POP-1 in posterior daughter cells in *efl-3* mutants that already experience low POP-1 levels can lead to premature differentiation (Fig. 4E). The transcriptional activator function of POP-1 requires a limited amount of POP-1, so it is likely that *efl-3* RNAi is not strong enough to perturb this function. The gains of seam cells were supressed in *efl-3* mutants treated with *egl-18* RNAi, indicating that *egl-18* is required for the seam cell gains. Notably, we found overexpression of *egl-18* in anterior differentiating daughters in *efl-3* mutants, which is known to result in ectopic seam cell fate maintenance and is associated with increased seam cell numbers (Gorrepati et al., 2013; Katsanos et al., 2017). The seam cell losses were found to be enhanced upon *egl-18* RNAi, possibly because in *efl-3* mutants there is simultaneous perturbation of *egl-18* and additional POP-1 targets promoting the seam cell fate. While changes in POP-1 levels can explain both seam cell gains and losses, our results demonstrate that absolute POP-1 levels alone cannot fully explain the loss of seam cells specifically at the L4 stage. We speculate that the sensitivity to changes in POP-1 activity at L4 may either stem from different thresholds required for POP-1 to act as a repressor and seam cell activator at L4 or, alternatively, from some additional sensitisation of seam cells at this developmental stage. Such sensitisation may link to changes in the abundance or distribution of Wnt signalling components, such as β-catenins, as animals progress through development. For example, the negative regulator PRY-1 has been reported to be more widely distributed in seam cells at the L4 stage in comparison to earlier developmental stages (Baldwin et al., 2016). Furthermore, we observed less pronounced asymmetry between anterior and posterior daughter cells at the L4 stage in wild type, which may point to stage-specific differences in Wnt regulation. Phenotypic sensitisation may ultimately depend on interactions between spatial patterning machinery and the heterochronic pathway defining temporal seam cell identities (Mallick et al., 2019; Ren and Zhang, 2010).

E2F-mediated regulation of proliferation and Wnt signalling are fundamental pathways in healthy development with important implications in diseases such as cancer (Kent and Leone, 2019; Xie et al., 2021; Zhan et al., 2017). However, the exact crosstalk between E2Fs and Wnt signalling is not well understood. One study recently reported that atypical E2F8 knockdown inhibits the Wnt signalling pathway in ovarian cancer cells (Zhang et al., 2023). Canonical E2F1 is also known to activate expression of axin 2, inhibiting Wnt signalling to promote cell death in mammalian cells (Hughes and Brady, 2005). E2F1 has been shown to activate the expression of the Wnt receptor Frizzled-1 in human osteoblasts to facilitate cell differentiation (Yu et al., 2013). Our findings add to these reports by providing a mechanistic link between EFL3/E2F7 and the NLK-Tcf/Lef pathway in the context of epidermal stem cell fate determination in *C. elegans*. Besides TCF, NLK phosphorylates many other substrates so a link between E2F7 and NLK may have broader implications for vertebrate and invertebrate development.

## MATERIALS AND METHODS

### *C. elegans* maintenance and genetics

All *C. elegans* strains in this study were maintained at 20°C on nematode growth medium (NGM) plates seeded with *Escherichia coli* OP50 (Brenner, 1974). A list of strains used in this study is provided in Table S3 and all strains are available upon request.

### Molecular cloning and transient transgenesis

To construct the seam cell CRE expression vector pHIN32, a *C. elegans* codon-optimised woCre recombinase gene was amplified from pDK56 with oligos Wcre_F and Wcre_R. The amplicon was inserted by Gibson Assembly into pDK70 containing the *srf-3i* promoter (Katsanos et al., 2021). pHIN32 was injected into *efl-3* floxed animals at 5 ng/μl with 5 ng/μl *myo-2::dsRed* as co-injection marker and 90 ng/μl BJ36 as carrier DNA.

To overexpress *lit-1* using the last intron of *arf-5* (*arf-5i*), *lit-1^d^* fragment was amplified from PHX7906 cDNA using the oligos (pes10)_lit-1_Fw and (unc54)_lit-1_Rv. The amplicon was inserted by Gibson Assembly into pIR5 to create pMFM20. pMFM20 was injected into JR667 at 20 ng/μl with 5 ng/μl *myo-2::dsRed* as co-injection marker and 85 ng/μl BJ36 as carrier DNA. A complete list of oligos and vectors used can be found in Table S3 and are available upon request.

### RNAi

RNAi by feeding was performed using *E. coli* HT115 expressing double-stranded RNA corresponding to the target gene or the control dsRNA plasmid (empty vector) as food source (Kamath et al., 2003). Bacteria cultures were grown overnight and 300 μl were seeded onto NGM plates containing 25 μg/ml ampicillin, 12.5 μg/ml tetracyclin and 1 mM IPTG. All RNAi treatments were performed during the postembryonic development by seeding eggs from the corresponding strain onto the RNAi plates. RNAi bacteria clones used in this study were from the commercially available Ahringer RNAi Library (Kamath and Ahringer, 2003) (Source Bioscience).

### Stable transgenesis and CRISPR genome editing

The endogenous copy of both *efl-3* and *lit-1* was tagged with codon-optimised GFP (288 aa) and the auxin-inducible degradation (AID) domain (45 aa) just before the stop codon at the C terminus using a CRISPR/Cas-9 approach (Suny Biotech). In both cases, a 9-aa 3xGAS linker was introduced between the protein of interest and GFP and a 6-aa 2xGAS linker between the GFP and the AID domain.

*loxP* sites flanking the *efl-3* endogenous locus (178 bp upstream of the transcription start site and 351 bp downstream of the 3′UTR) were introduced by injecting JR667 animals with 0.25 ng/μl protein Cas9, 2.25 μM tracrRNA, 2.38 μM of 5′_efl-3_crRNA and 3′_efl-3_crRNAs, 5.5 μM of 5′ and 3′ repair templates and 5 ng/μl *myo-2::dsRed*. crRNAs targeted 241 bp upstream of the TSS and 298 bp downstream of the TES. The 5′ and 3′ *efl-3* repair templates each had 50 bp homology flanking the Cas9 cut site and included a *loxP* sequence and a ClaI cut site. The 5′ template also contained M13uni_43 sequence, while the 3′ template contained M13rev_49 sequence. Transgenic animals with red pharynx were genotyped using the oligos efl-3_5p_F, R and efl-3_3p_F, R for 5′ and 3′ modification, respectively. The PCR product was digested with ClaI restriction enzyme to confirm successful homologous repair.

To delete the predicted EFL-3-binding region of the *lit-1* locus, PHX7906 animals were injected with 0.25 ng/μl protein Cas9, 2.25 μM tracrRNA, 2.38 μM of 5′_lit-1_crRNA and 3′_lit-1_crRNA and 5 ng/μl *myo-2::dsRed*. crRNAs targeted a 2743 bp long region inside the *lit-1d* first intron. Transgenic animals with red pharynx were screened for the presence of the deletion using the oligos lit-1idel_Fw and lit-1idel_Rv. Deletion of the EFL-3 binding region was confirmed by sequencing.

The single-copy woCRE strain was generated with the MosSCI method using animals of the EG8080 strain with a Mos1 transposon insertion on chromosome III (*oxTi444*) (Frøkjær-Jensen et al., 2014). pHIN32 was injected at 50 ng/μl together with 50 ng/μl of pCFJ601 (Mos1 transposase), 10 ng/μl of pMA122 (heat-shock-inducible *peel-1* toxin) and 10 ng/μl, 2.5 ng/μl and 5 ng/μl of the co-injection markers pGH8 (*rab-3::mCherry*), *myo-2::dsRed* and *myo-3::mCherry*, respectively. After injection, animals were kept at 25°C until they were starved. Heat shock was performed at 33°C for 3.5 h and the plates were allowed to recover for 3 h at room temperature before ‘reverse chunking’. The next day the top of the laws on each chunk was screened for normal roaming animals with the absence of co-injection markers. Homozygous lines were confirmed molecularly for the presence of single-copy transgenes using oligos NM3880 and NM3884. A complete list of oligos can be found in Table S3.

### Phenotypic analysis and microscopy

For phenotypic characterisation, animals were anesthetised on 3% agarose pads containing 100 μM sodium azide (NaN_3_), secured with a coverslip and visualised under an AxioScope A1 (Zeiss) upright epifluorescence microscope with an LED light source with a RETIGA R6 camera controlled by Ocular software. Seam cell number was scored in one lateral side of early adult animals containing the *SCM::GFP* marker (Koh and Rothman, 2001). This seam cell marker (SCM) includes the fourth intron of the *arf-5* gene, which is sufficient on its own to drive expression in seam cells. Visualisation of the seam cell membrane was achieved with *wrt-2::GFP::PH; wrt-2::GFP::H2B* markers and animals were scored at 30 h post-bleaching for late L2 stage, at 40 h post-bleaching for late L3 stage and 48 h post-bleaching for late L4 stage.

Protein reporter images were acquired using a Leica SP8 inverted confocal microscope with a 63×/1.4 oil DIC objective controlled by LAS X software. Animals were mounted in 5% agarose pads containing 100 μM NaN_3_ and secured with a coverslip. To visualise L2 symmetric and asymmetric divisions animals were observed at 26 h post-bleaching. To visualise L3 asymmetric divisions, animals were synchronised by egg laying over a period of 2 h and observed after 35 h. Images were acquired in a single plane that provided the best focus for the seam cell nuclei. Fluorescence intensity for EFL-3::GFP, LIT-1::GFP, EGL-18::mNG and GFP::POP-1 was quantified using Fiji macros. For Fig. S8D,E, a region of interest (ROI) of the same size was used to quantify images at the L2 and L4 stages. The data were normalised (rescaled) to [0,1] using the formula:

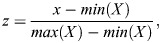
where *X* is the data, *x* is a datapoint and *z* is the corresponding rescaled datapoint. Image editing was performed using straightening tool and stitching macro from Fiji (Preibisch et al., 2009).

For DAPI intensity quantification, 23 *z*-stack slices of 0.8 μm were acquired 25 h post-bleaching from fixed animals using the 100× objective of an epifluorescence Ti-eclipse microscope (Nikon) and DU-934 CCD-17291 camera (Andor Technology) operated by Nikon NIS Elements software. The apical junction marker AJM-1::GFP was used to determine the developmental stage by distinguishing when anterior cells round up to differentiate. ROIs were manually drawn around the seam cell nuclei. DAPI fluorescence intensity was quantified by measuring the integrated density and area of the manually drawn ROIs in single sections where the DAPI signal was in focus. Three background readings were obtained from the area surrounding the seam cells. To avoid biases arising from variations in DNA compaction and nuclear size, we calculated the corrected total cell fluorescence [CTCF=ROI integrated density−(ROI area×average of three background readings)], which gives total DAPI intensity instead of mean fluorescence. Daughter cells from a symmetric division were included in the quantification as control.

To analyse developmental speed and division synchrony, animals expressing the membrane were fixed at 25 h post-bleaching. For the division asynchrony analysis, lineages were classified into three different categories based on the expression of AJM-1::GFP: dividing; asymmetric division completed early; and asymmetric division completed late.

### smFISH

Animals were synchronised by bleaching and were fixed at the appropriate stage using 4% formaldehyde for 45 min and permeabilised with 70% ethanol. Samples were then incubated with a pool of 24-48 oligos labelled with Quasar 670 (Biosearch Technologies) for 16 h. Imaging was performed using the 100× objective of an epifluorescence Ti-eclipse microscope (Nikon) and DU-934 CCD-17291 camera (Andor Technology) operated using the Nikon NIS Elements Software. Twenty-three *z*-stack slices of 0.8 μm were acquired in DAPI, Cy5 and GFP channels for each animal. Spot quantification was performed using MATLAB (MathWorks) as previously described (Raj et al., 2008). Z-slices containing spots were maximum projected to give a single image, inverted and merged to the GFP channel using ImageJ (NIH). A complete list of the smFISH probes used in this study is provided in Table S3.

### RNA extraction and cDNA preparation

Animals from the PXH7906 strain were collected at L2 stage and total RNA was extracted using TRIzol reagent (Invitrogen) and isopropanol/ethanol precipitation. NanoDrop quantification and gel electrophoresis was used to assess the quantity and quality of RNA. Two milligrams of RNA was subjected to DNase (Promega) treatment and cDNA was synthesised using Superscript IV (Invitrogen) with Oligo(dT) primers as per the manufacturer's instructions.

### Extraction and amplification of methylated DNA

For NanoDam experiments, animals were synchronised by bleaching and grown for three generations in *E. coli dam*^−/−^ *dcm*^−/−^ bacteria (New England Biolabs, C2925). Two biological replicates were grown in parallel for strains carrying the NanoDam transgenes with and without *efl-3:gfp* for C-terminal and N-terminal Dam:GFP-nanobody configurations. Animals were collected after 48 h, extensively washed five times in M9 buffer and stored at −20°C. Purification and amplification of methylated DNA was performed as previously described (Katsanos et al., 2021). Extracted gDNA was digested with DpnI and double-stranded adaptors were ligated using T4 DNA ligase. Then, DNA fragments were digested with DpnII and amplified using MyTaq polymerase. Amplicons were purified using QIAquick PCR purification kit and adaptors were removed by AlwI digestion followed by another purification. Library preparation and Next Generation Sequencing on an Illumina HiSeq 4000 platform was performed by GENEWIZ.

### NanoDam signal profiles, peak calling, and gene assignment

FASTQ files representing paired-end reads for each sample and replicate were processed using the damidseq_pipeline (v.1.5.3) (Marshall and Brand, 2015). Reads were mapped to the *C. elegans* WBcel235 genome assembly using bowtie2 (v.2.4.5) and scores were calculated per GATC fragment of the genome. Pairwise genome-wide calculations of log_2_(EFL-3:GFP; Dam:GFP-nanobody/Dam:GFP-nanobody) ratio scores per GATC fragment of the genome for each Dam:GFP-nanobody configuration were performed from bam files to produce bedGraph signal files that were normalised by quantile normalisation and arithmetically averaged into a single signal profile. Peak calling was performed using the perl script find_peaks (available at https://github.com/owenjm/find_peaks) with FDR<0.05. Statistically significant peaks were assigned to genes using UROPA (Kondili et al., 2017) with Caenorhabditis_elegans.WBcel235.106.gtf as the genome annotation file. Peaks were assigned to genes when their centre coordinate was positioned up to 5 kb upstream of a gene start site or 2 kb downstream of the end site. Visualisation of signal tracks and other genomic features was performed using Integrative Genomics Viewer.

### Aggregation plots and heatmaps of signal localisation

Signal aggregation plots and peak localisation heatmaps were generated using the SeqPlots GUI application (Stempor and Ahringer, 2016). Signal and peaks were visualised across genes containing peaks up to 5 kb upstream of a gene start site or 2 kb downstream of the end site. Genes were plotted as ‘anchored’ features, whereby the TSS and the TES are fixed in the *x*-axis and the genic sequence is adjusted to a pseudo-length of 2 kb. In aggregation plots, signal is averaged in 10 bp bins and the *y*-axis represents deviations from the average signal across the plotted region (*z*-scores). In heatmaps, each coloured line represents the position of a statistically significant peak.

### Assessment of overlap between statistically significant peaks

To calculate overlapping statistically significant peaks between the C-terminal and N-terminal Dam:GFP-nanobody configurations, BEDTools intersect tool was used (Quinlan and Hall, 2010). To test whether overlaps where statistically significant, Monte Carlo simulations were performed using OLOGRAM (Ferré et al., 2019).

### Motif identification analysis

Motif analysis was performed using MEME (https://meme-suite.org/meme/). FIMO was used to scan for individual matches of specific motifs provided (Grant et al., 2011). These motifs were downloaded from the JASPAR database (https://jaspar2022.genereg.net).

### Gene set enrichment analysis

Gene sets identified in this study were assessed for enriched GO terms using WormCat (Holdorf et al., 2020). A *P*-value of <0.005 was used for significance threshold and plots show −log__10_(_pvalue).

### Quantification and statistical analysis

Graphic representation and statistical analysis were performed using R. Error bars used in all graphs represent the s.d. Heatmaps represent the relative frequency of seam cell defects per cell lineage, which was calculated by dividing the total number of gains or losses in a cell lineage by the total number of animals analysed. An unpaired, two-tailed *t*-test or χ^2^ test was used to evaluate significance as indicated in the figure legends. Results were considered statistically significant when *P*<0.05. Asterisks in figures indicate corresponding statistical significance as follows: *P<0.05; **P<0.01; ***P<0.005; ****P<0.001.

## Supplementary Material

10.1242/develop.204546_sup1 Supplementary information

Table S1. List of statistically significant EFL-3 NanoDam peaks.

Table S2. List of putative EFL-3 target genes in seam cells.

Table S3. List of C. elegans strains, smFISH probes, oligos and vectors used in this study.
