# Supplementary material for: EFL-3/E2F7 modulates Wnt signalling by repressing the Nemo-like kinase LIT-1 during asymmetric epidermal cell division in Caenorhabditis elegans
Source: Development. 2025 Mar 3;152(5):DEV204546. doi: 10.1242/dev.204546 (PMC11925398; doi:10.1242/dev.204546)
Supplement: Supplementary information [file develop-152-204546-s1.pdf]

A

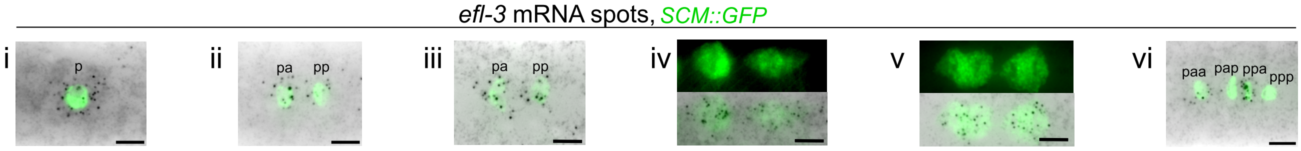

B

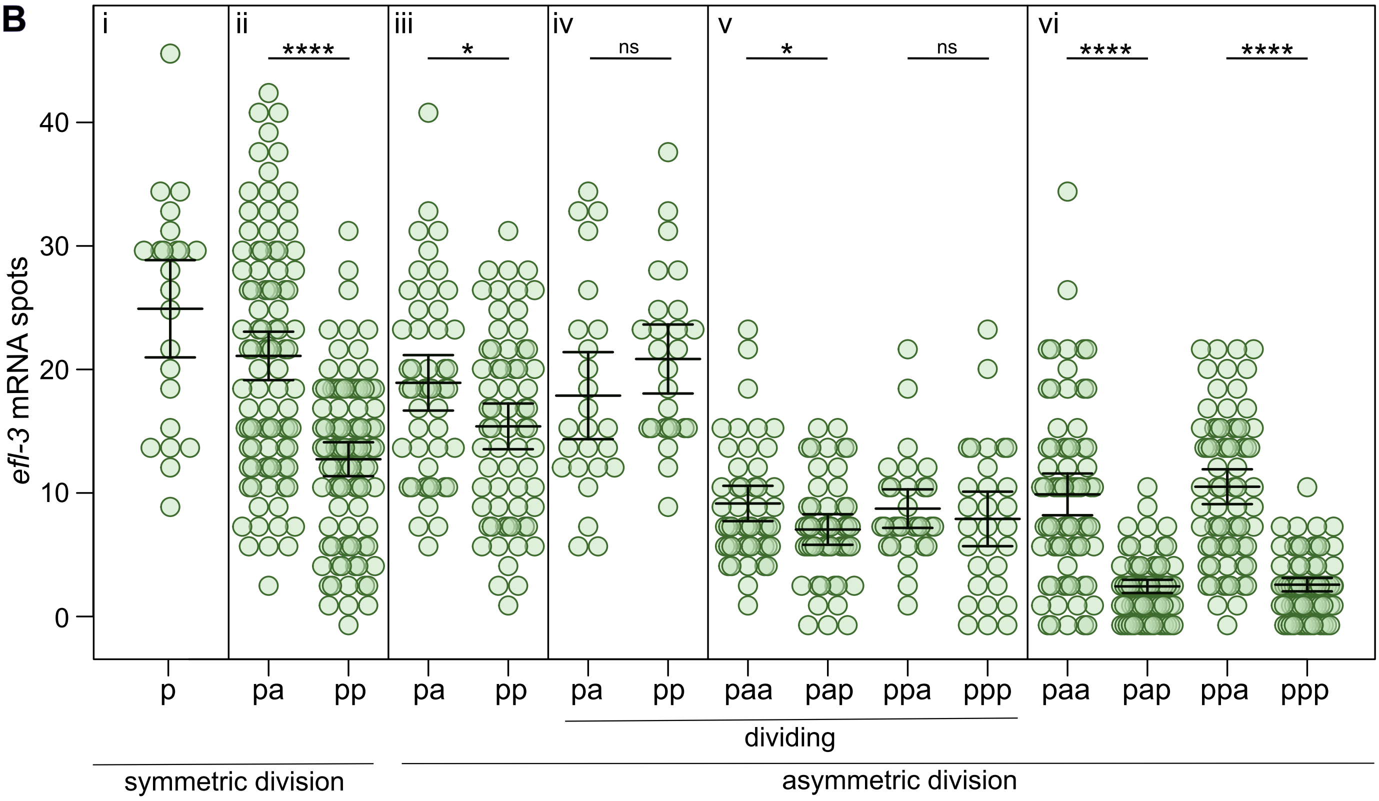

C

Stage	i	ii		iii		iv		v				vi			
Cell	p	pa	pp	pa	pp	pa	pp	paa	pap	ppa	ppp	paa	pap	ppa	ppp
% of cells with intense spots	0 %	11 %	1 %	4 %	4 %	0 %	4 %	0%	2 %	3 %	7 %	1%	0 %	4 %	0 %
Total number of cells	23	93	93	48	81	25	27	47	47	30	31	71	71	71	71
Chi-Square test	N/A	p < 0.005		ns		ns		ns		ns		ns		ns	

D

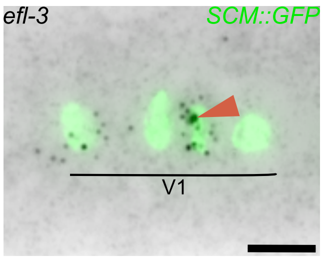

Development • Supplementary information

Fig. S1. Characterisation of EFL-3 expression via smFISH. (A) Analysis of *efl-3* expression in the V1-V4 lineages from late L1 (i), after L2 symmetric division (ii), before L2 asymmetric division (iii), metaphase (iv) of L2 asymmetric division, anaphase-telophase of L2 asymmetric division (v), and after the L2 asymmetric division (vi). **(B)** Quantification of *efl-3* mRNA spots in V1-V4 lineages in each of the developmental stages shown in (A); n ≥ 23 cells per condition; pa, paa and ppa are anterior daughter cells and pp, ppa and ppp are posterior daughter cells. Error bars show the mean ±standard deviation. Black stars indicate statistically significant differences in the mean with a two-tailed t-test. * $p<0.05$, **** $p<0.001$. **(C)** Percentage of cells containing intense nuclear spots that may correspond to active transcription events. Animals were analysed in each of the developmental stages shown in (A). Chi-square *p*-value was calculated between anterior (pa, paa, ppa) and posterior (pp, pap, ppp) lineages for each developmental stage, respectively. **(D)** Representative image of *efl-3* intense spot (red arrowhead in the anterior cell) after the L2 asymmetric division. In A and D seam cell nuclei are labelled with *SCM::GFP* and scale bars are 5 μm.

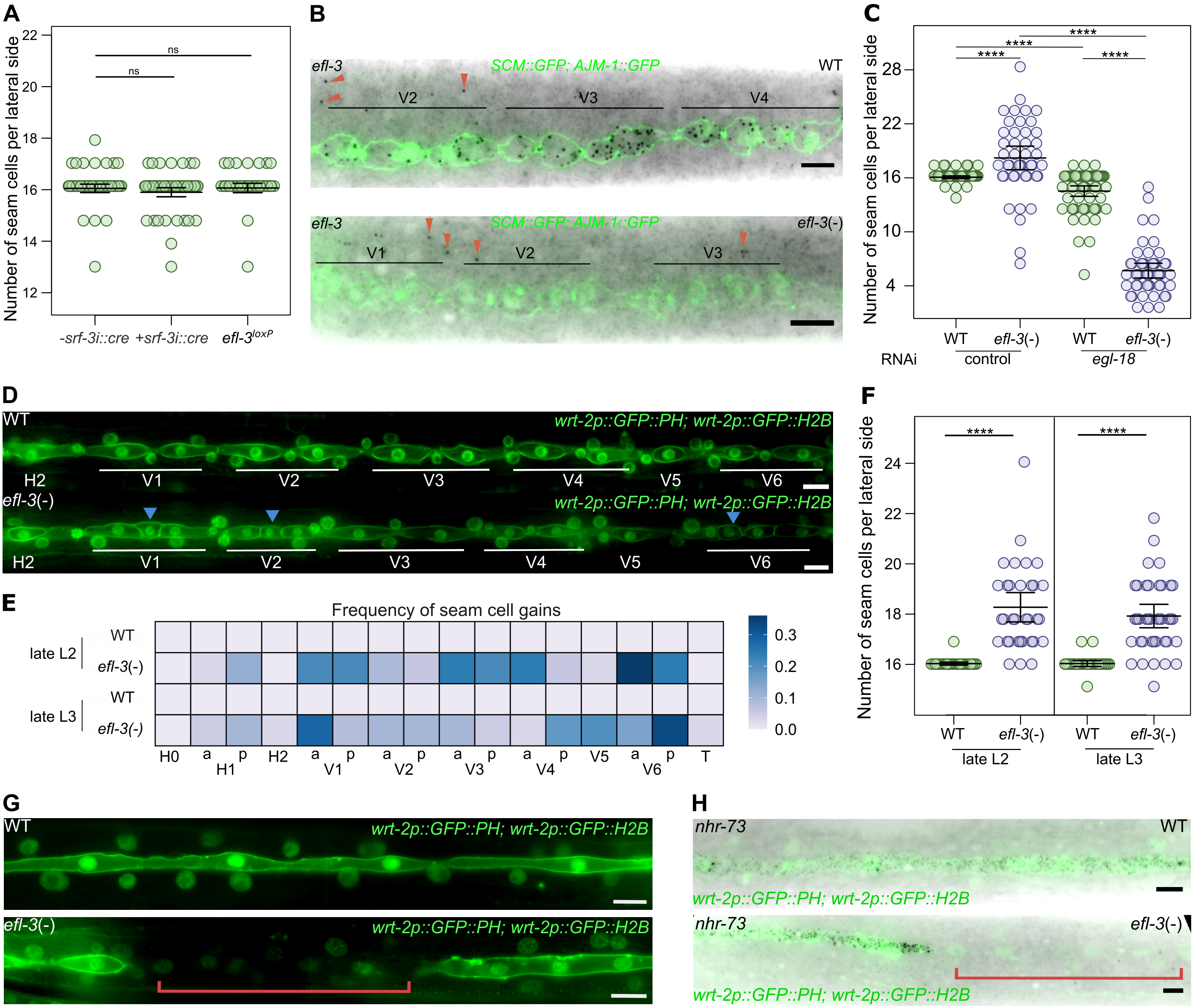

Development • Supplementary information

Fig. S2. Phenotypic characterisation of *efl-3* mutants. (A) Seam cell counts in wild-type *(-srf-3::cre*), animals expressing the CRE recombinase in the seam cells (+*srf- 3::cre*) and animals with *efl-3* locus floxed, n ≥ 30 animals per condition. **(B)** Representative images of *efl-3* smFISH following the L2 asymmetric division in wild-type (WT) and in *efl-3* mutant animals (*efl-3*(-)). Seam cell nuclei are labelled using *SCM::GFP* and the apical junction marker *ajm-1p::ajm-1::GFP. efl-3*(-) animals don't show *efl-3* mRNA spots in the seam cells. Red arrowheads point to *efl-3* mRNA spots in the hypodermis. **(C)** Seam cell counts in wild-type and *efl-3* mutant animals treated with control and *egl-18* RNAi. Error bars show the mean ±standard deviation, n ≥ 46 animals per condition. **(D)** Representative images of seam cells at the late L2 stage in wild type and in *efl-3* mutant animals. Seam cells are labelled with the epidermal marker *wrt-2p::GFP::PH; wrt-2p::GFP::H2B*. Blue arrowheads indicate anterior daughter cells that failed to differentiate following asymmetric division. **(E)** Heatmaps showing frequency of seam cell gains per cell lineage at the late L2 and late L3 stages in wild-type and in *efl-3* mutant animals. No seam cell losses were observed in any of these stages, n ≥ 30 animals per condition, a = anterior lineage, p = posterior lineage. **(F)** Seam cell counts in wild-type and *efl-3* mutant animals at the late L2 and late L3 stages. *efl-3* mutants present a significant increase in the average number of seam cells compared to wild-type, n ≥ 30 animals per condition. **(G)** Representative images of the seam cells at the L4 stage in wild-type (WT) and in *efl-3* mutant animals (*efl-3*(-)). Seam cells are labelled with the epidermal marker *wrt-2p::GFP::PH; wrt-2p::GFP::H2B*. **(H)** Representative *nhr-73* smFISH images at L4 stage in wild-type (WT) and in *efl-3* mutant animals (*efl-3*(-)). In G and H, *efl-3* mutants present gaps in the seam cell line indicated with a red bracket.

Development • Supplementary information

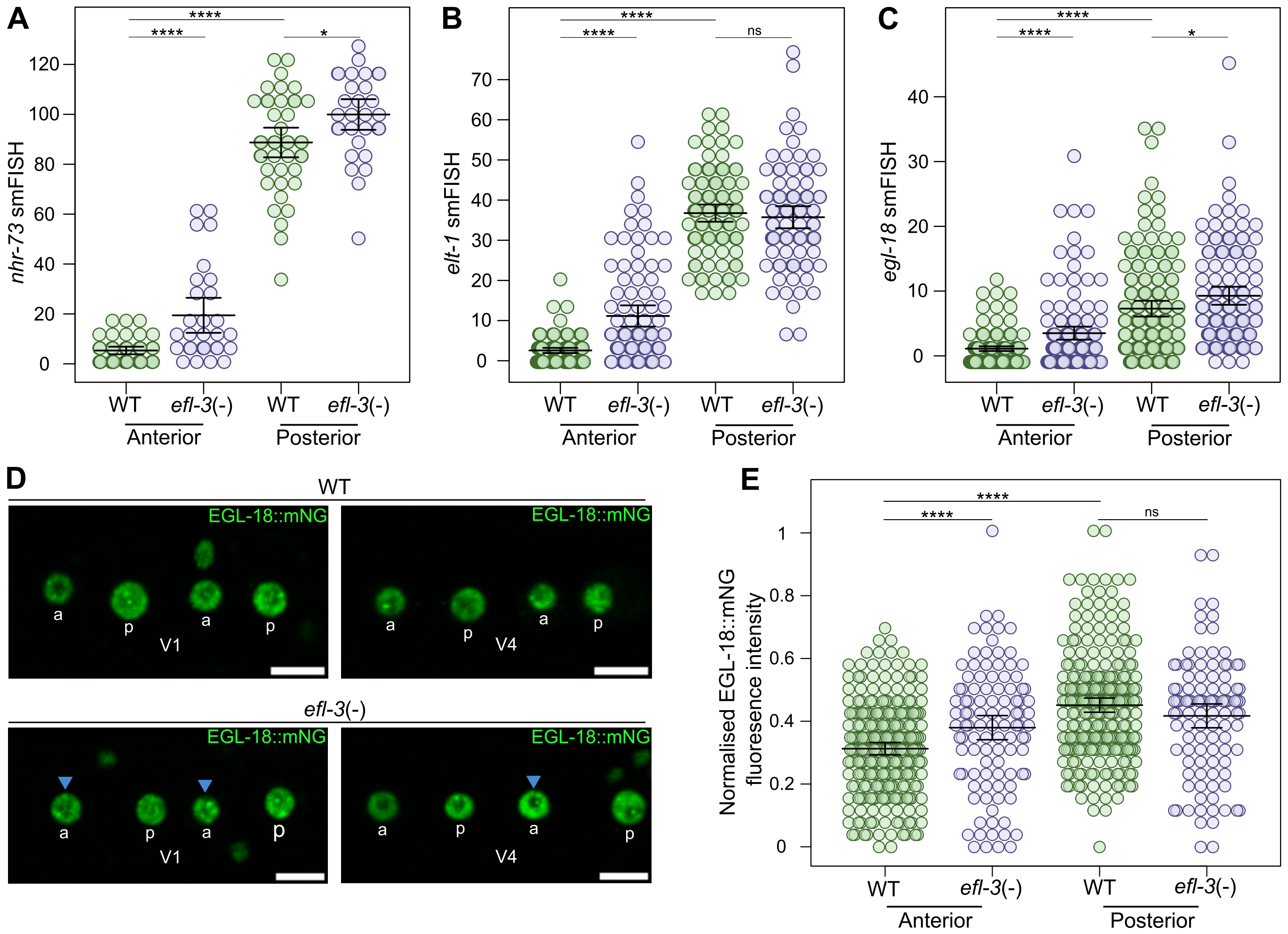

A
B
C
nhr-73 smFISH
elt-1 smFISH
egl-18 smFISH
WT
efl-3(-)
Anterior
Posterior
****
*
ns
D
E
EGL-18::mNG
V1
V4
a
p
Normalised EGL-18::mNG fluoresence intensity

Fig. S3. Analysis of seam cell marker expression in *efl-3* mutants. (A-C) Quantification of mRNA spots in wild-type (WT) and *efl-3* mutant animals (*efl-3*(-)) following the L2 asymmetric division for *nhr-73* (A), *elt-1* (B) and *egl-18* (C). n ≥ 30 cells in A, n ≥ 86 cells in B and n ≥ 122 cells in C. **(I)** Representative images of EGL-18::mNeonGreen following the L2 asymmetric division in wild-type (WT) and in *efl-3* mutant animals (*efl-3*(-)). Blue arrowheads indicate representative anterior daughter cells with higher expression of EGL-18::mNeonGreen. **(J)** Quantification of EGL-18::mNeonGreen following the L2 asymmetric division in wild-type (WT) and in *efl-3* mutant animals (*efl-3*(-)); n ≥ 100 cells per condition. Error bars in A, B, C and E show the mean ±standard deviation. * p<0.05 and **** p<0.001 with a two-tailed t-test. Scale bars are 5 μm D.

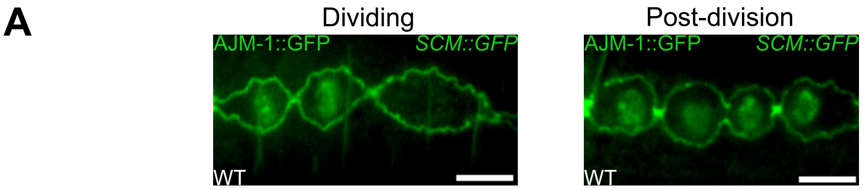

	V1		V2		V3		V4	
	WT	*efl-3*(-)	WT	*efl-3*(-)	WT	*efl-3*(-)	WT	*efl-3*(-)
Dividing	23%	9%	0%	9%	18%	9%	5%	0%
Post-division	77%	91%	100%	91%	82%	91%	95%	100%
Chi-square	p = 0.21		p = 0.52		p = 0.38		p = 0.97	

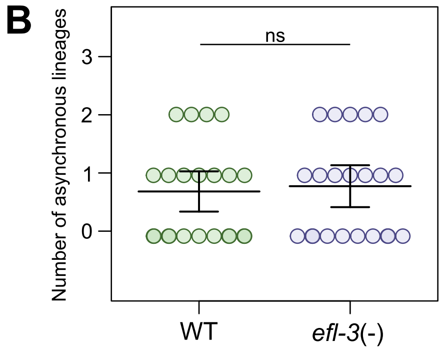

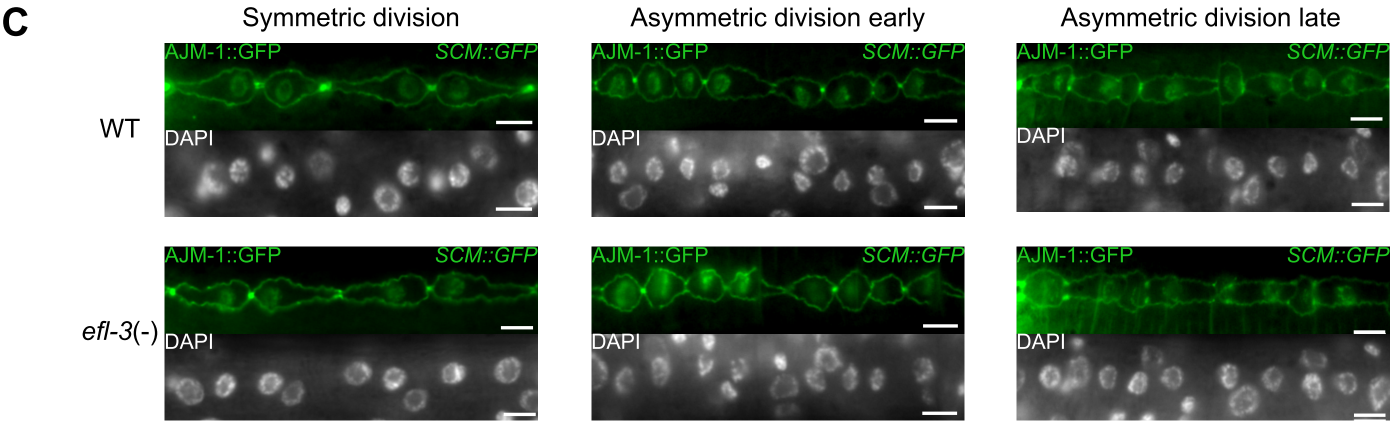

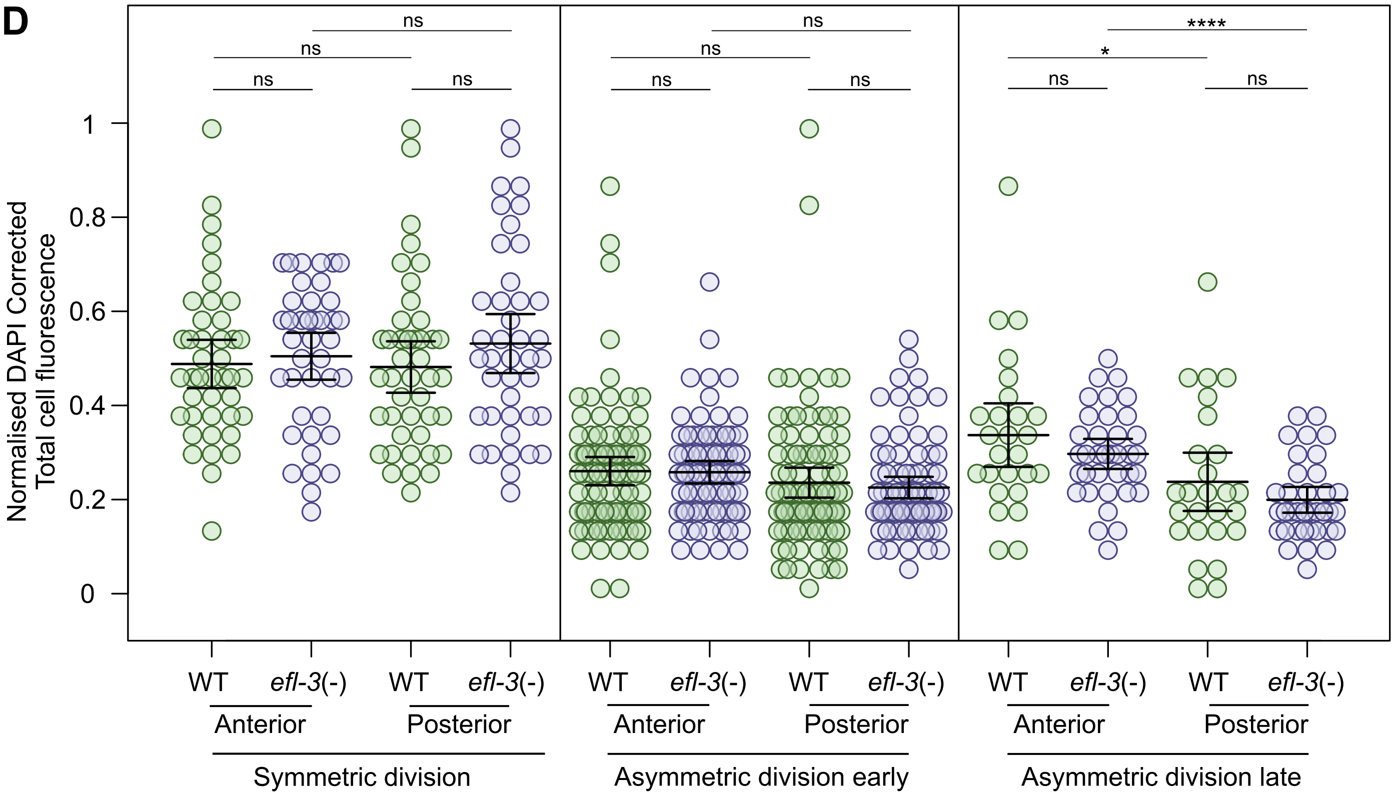

Fig. S4. Analysis of developmental speed, division synchrony and endoreduplication in *efl-3* mutants. (A) Percentage of cases where one V1-V4 cell still undergoes L2 asymmetric division ("Dividing") or both cells have completed the L2 asymmetric division ("Post-division") at 25 hours post-bleaching in WT and tissue-specific *efl-3* mutants (*efl-3*(-)); n = 22 animals per condition. **(B)** Number of asynchronous lineages per animal in WT and *efl-3* tissue-specific mutants (*efl-3*(-)) at 25 hours post-bleaching; n = 22 animals per condition. **(C)** Representative images *of ajm-1p::ajm-1::GFP; SCM::GFP* and DAPI staining following the L2 symmetric division, and the L2 asymmetric division early and late in wild-type (WT) and in *efl-3* mutant animals (*efl-3*(-)). **(D)** Quantification of DAPI Corrected Total Cell Fluorescence (CTCF) intensity per median section of the nucleus in anterior and posterior daughter cells following the L2 symmetric division, and the L2 asymmetric division early and late in wild-type (WT) and in *efl-3* mutant animals (*efl-3*(-)); n ≥ 36 cells per condition. Error bars in B and D show the mean ±standard deviation. * p<0.05 and **** p<0.001 with a two-tailed t-test. Scale bars are 5 μm in A and C.

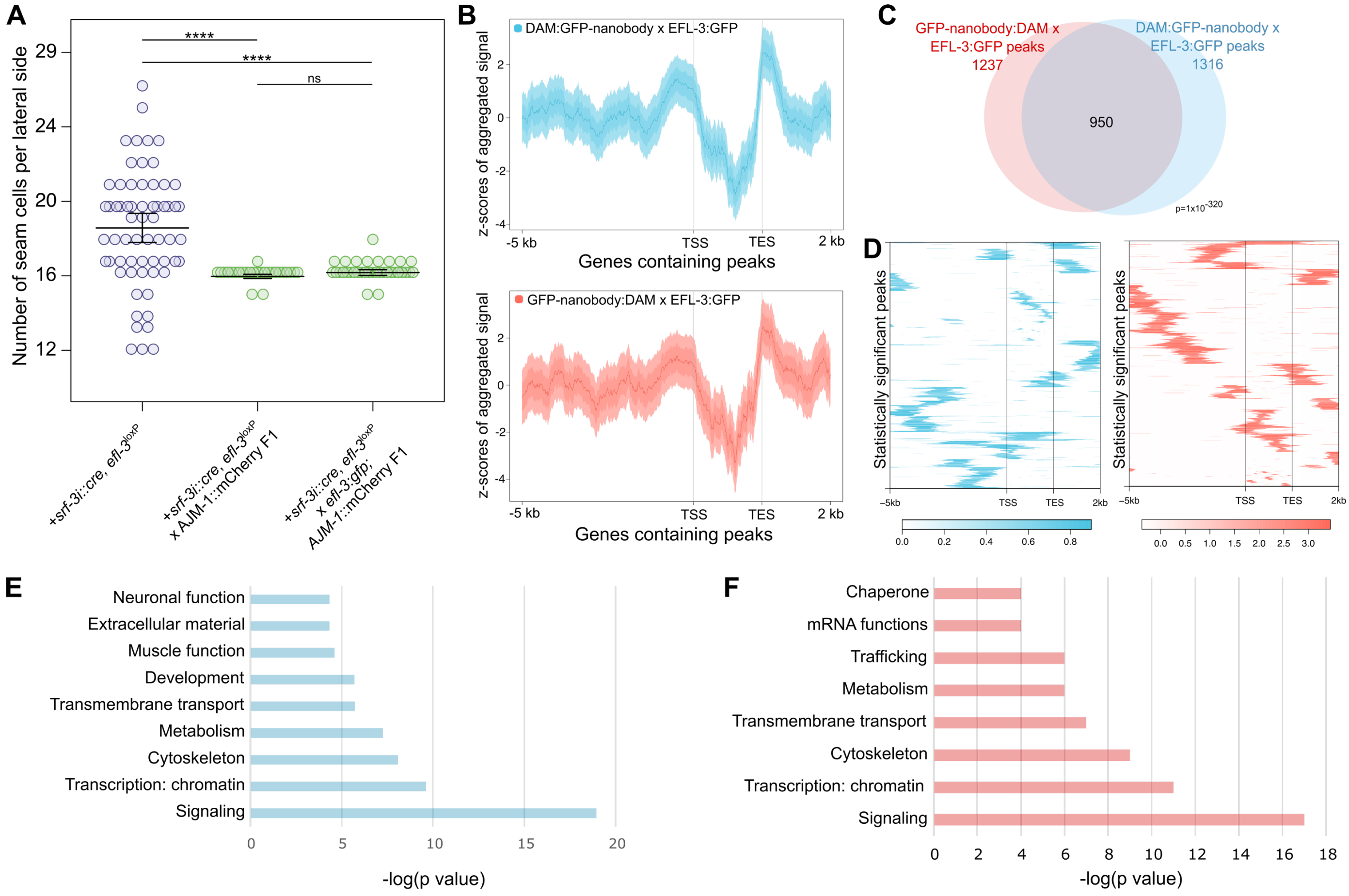

Figure S5

Development • Supplementary information

Fig. S5. NanoDam analysis of EFL-3::GFP binding. (A) Seam cell counts in *efl-3* mutants, and in the F1 progeny of *efl-3* mutants crossed with WT or with *efl-3::gfp* animals. Rescue of the phenotype demonstrates the functionality of the EFL-3::GFP fusion. Cross progeny was identified via expression of the apical junction marker *ajm-1p::mCherry*; n ≥ 32 animals per condition; **** $p<0.001$ with a two-tailed t-test. **(B)** Aggregation plots of profiling data generated by EFL-3 NanoDam showing average enrichment scores in 10-bp bins for regions of equal length across all genes containing statistically significant peaks. Enrichment is seen upstream and downstream of gene regions. Plots show 5 kb upstream of the transcription start site (TSS) and 2 kb downstream of the transcription end site (TES). Gene bodies are condensed into a 2 kb pseudo-length. Shaded areas represent 95% confidence intervals. **(C)** Venn diagram of all call peaks between EFL-3 C- and N-terminal NanoDam. P value was calculated by Monte Carlo simulations for significance of overlap between datasets. **(D)** Heatmaps representing the hierarchically clustered localization and enrichment score of all statistically significant peaks (FDR < 0.05) within 5 kb upstream and 2 kb downstream of genes containing peaks. (E-F) Plots of significantly enriched terms from GO-term analysis for C- (E) and N-terminal (F) EFL-3 NanoDam-identified targets.

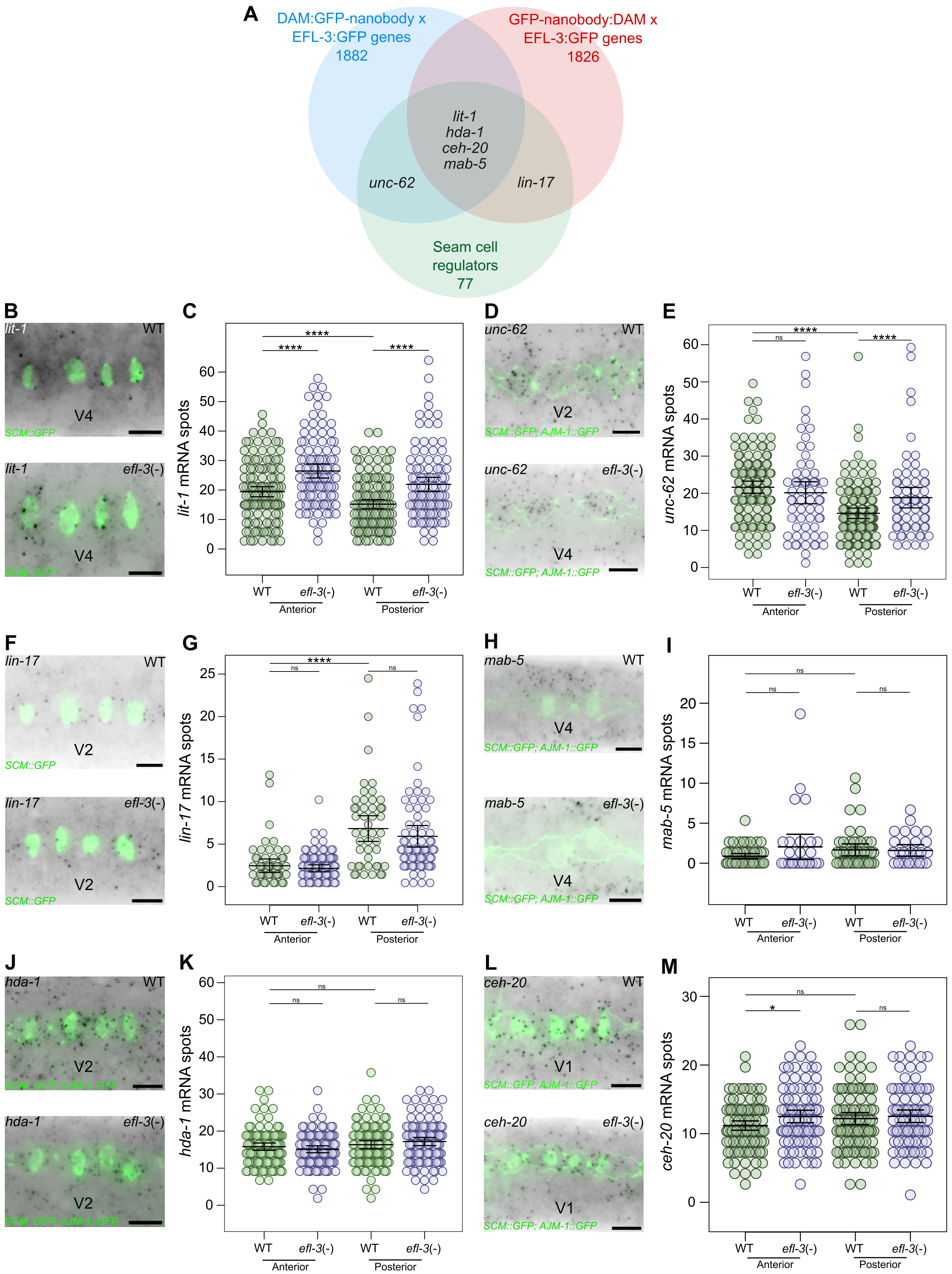

Figure S6

Development • Supplementary information

Fig. S6. smFISH expression analysis of EFL-3 putative target genes. (A) Venn diagram representing the overlap between EFL-3 putative target genes identified with C-terminal (blue) and N-terminal Dam:GFP-nanobody configuration (red) and a list of genes known to participate in seam cell development based on the literature. **(B-M)** Representative smFISH images and mRNA spot quantification in WT and *efl-3* mutants (*efl-3*(-)) for *lit-1* (B-C), *unc-62* (D-E), *lin-17* (F-G), *mab-5* (H-I), *hda-1* (J-K) and *ceh-20* (L-M). mRNA spots were quantified in V1-V4 lineages after the L2 asymmetric division for B-G and J-M, and before the L2 asymmetric division for H-I. In B, D, F, H, J, and L scale bars are 5 μm and seam cell nuclei are labelled with *SCM::GFP*. In D, H, J and L the seam cell membrane is labelled with *ajm-1p::ajm-1::GFP*. In C, E, G, I, K and M error bars show the mean ±standard deviation. * $p<0.05$, **** $p<0.001$ with a two-tailed t-test. $n \geq 100$ for C, $n \geq 44$ for E, $n \geq 46$ for G, $n \geq 28$ for I, $n = 102$ for K and $n \geq 102$ for M; n = cells per condition.

Development • Supplementary information

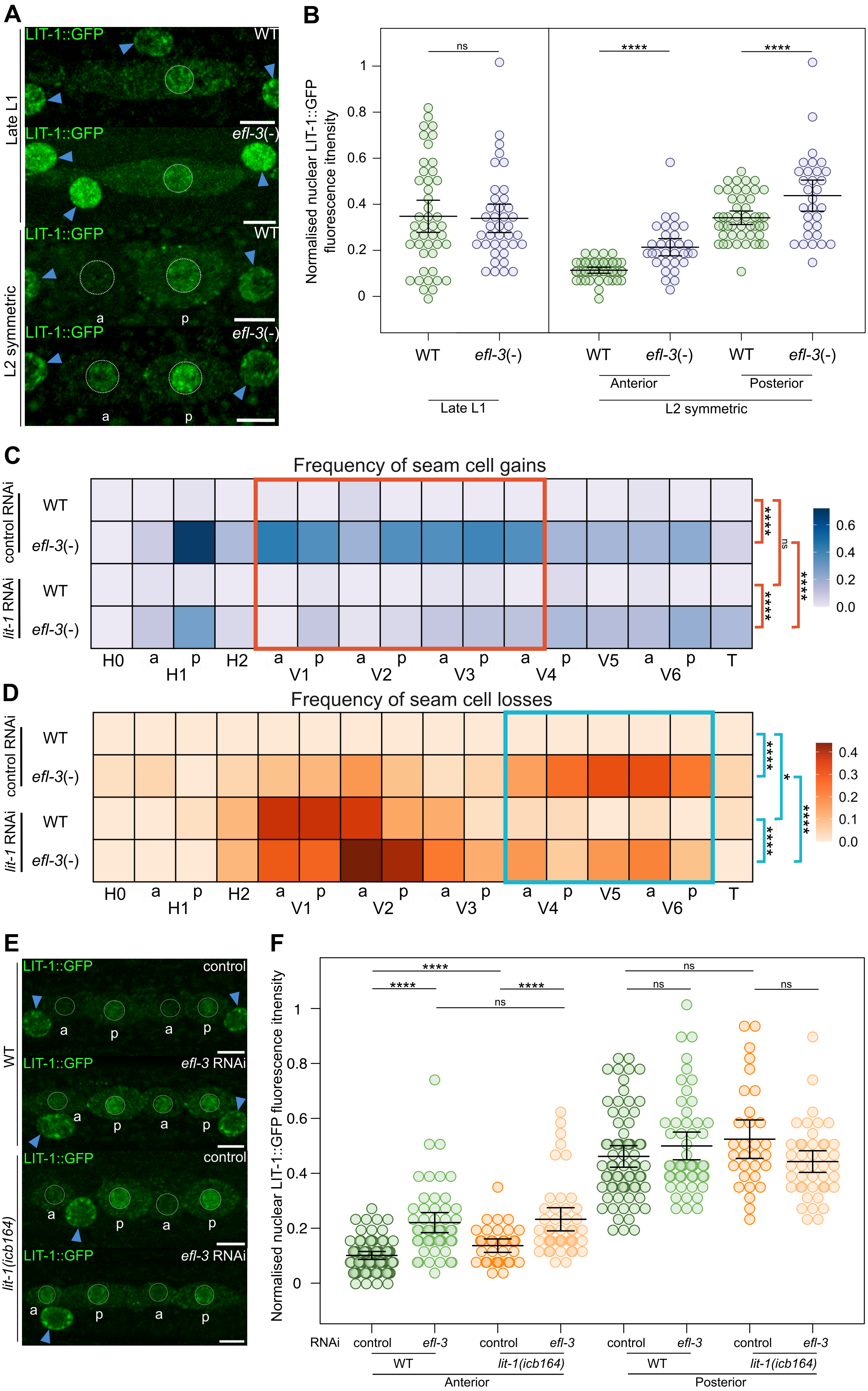

Development • Supplementary information

Fig. S7. EFL-3 regulates LIT-1 levels and *lit-1* downregulation reduces the gains and losses of seam cells observed in *efl-3* mutants. (A) Representative images of LIT-1::GFP at late L1 stage and following the L2 symmetric division in wild-type (WT) and *efl-3* mutant animals (*efl-3*(-)) **(B)** Quantification of LIT-1::GFP fluorescence intensity in the nuclei of V1-V4 seam cells at late L1 and after the L2 symmetric division. LIT-1::GFP is significantly increased in anterior and posterior daughter cells in *efl-3* mutant animals (*efl-3*(-)) compared to wild-type animals (WT*)* after the L2 symmetric division; n ≥ 31 cells per condition. **(C-D)** Heatmaps showing frequency of seam cell gains **(C)** and losses **(D)** per cell lineage at the end of postembryonic development in wild-type (WT) and in *efl-3* mutant animals (*efl-3*(-)) treated with control and *lit-1* RNAi. Chi-square test was performed for the pooled cell lineages highlighted in the red square in C and blue square in D. * $p<0.05$, **** $p<0.001$. In C and D, n = 50 animals per condition. **(E)** Representative images of LIT-1::GFP following the L2 asymmetric division in wild-type and *lit-1(icb164)* animals treated with control and *efl-3* RNAi*.* **(F)** Quantification of LIT-1::GFP fluorescence intensity in the nuclei of V1-V4 seam cells following the L2 asymmetric division. LIT-1::GFP is significantly increased in anterior daughter cells in *efl-3* RNAi-treated animals compared to control animals. Animals containing *lit-1(icb164)* CRISPR deletion didn't show further increase in LIT-1::GFP fluorescence intensity in anterior daughter cells compared to wild-type when treated with *efl-3* RNAi; n ≥ 35 cells per condition. In A and E, seam cell nuclei are circled in white, hypodermal nuclei are labelled with blue arrowheads, a = anterior daughter cell and p = posterior daughter cell and scale bars are 5 μm. Error bars in B and F show the mean ±standard deviation. **** $p<0.001$ with a two-tailed t-test.

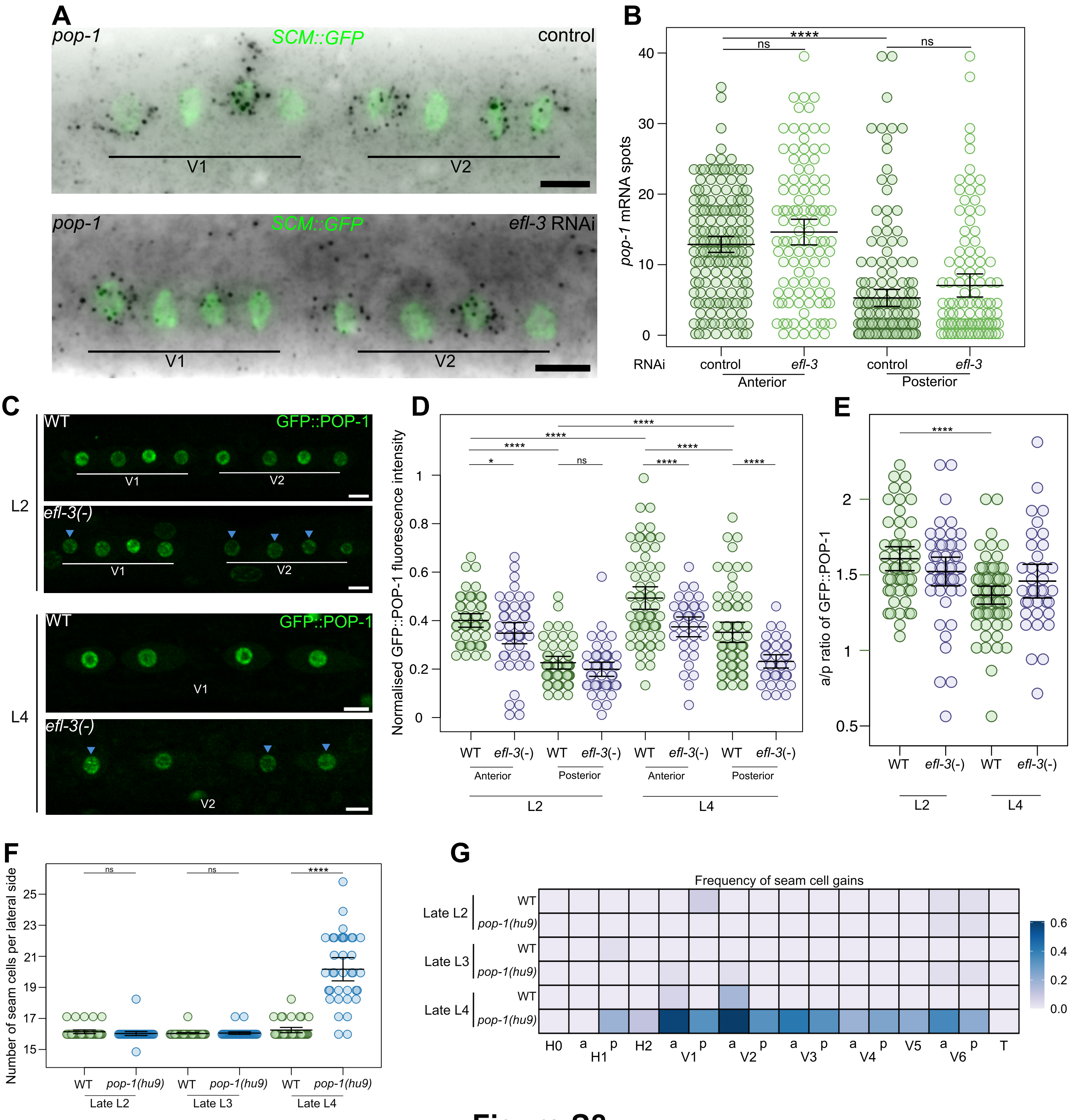

Figure S8

Development • Supplementary information

Fig. S8. *efl-3* mutants show changes in POP-1 nuclear distribution at L2 and L4 stage. (A) Representative *pop-1* smFISH images after the L2 asymmetric division upon *efl-3* RNAi compared to control treatment. Seam cells are labelled using *SCM::GFP*. **(B)** Quantification of *pop-1* mRNA spots in V1-V4 lineages in anterior and posterior daughter cells following the L2 asymmetric division in the conditions of A; n ≥ 108 cells per condition. **(C)** Representative images of GFP::POP-1 following the L2 and L4 asymmetric divisions in tissue-specific *efl-3* mutant animals (*efl-3*(-)) versus wild-type (WT). **(D)** Quantification of GFP::POP-1 fluorescence intensity following the L2 and L4 asymmetric divisions in tissue-specific *efl-3* mutant animals (*efl-3*(-)) versus wild type (WT); n ≥ 36 cells per condition. * $p<0.05$ and **** $p<0.001$ with a two-tailed t-test. Note that changes in POP-1 expression at L2 follow the same trend as in Figure 4B where sample size is larger. **(E)** Anterior-posterior GFP::POP-1 ratios following the L2 and L4 asymmetric divisions in tissue-specific *efl-3* mutant animals versus wild type, n ≥ 36 a-p pairs per condition. **** $p<0.001$ with a two-tailed t-test. **(F)** Seam cell counts in wild-type (WT) and *pop-1(hu9)* mutant animals at the late L2, late L3 and late L4 stages. At late L4, *efl-3* mutants present a significant increase in the average number of seam cells compared to wild type, n = 36 animals per condition. **(G)** Heatmap showing frequency of seam cell gains per cell lineage at the late L2, late L3 and late L4 stages in wild-type (WT) and in *pop-1(hu9)* mutant animals., n = 36 per condition, a = anterior lineage, p = posterior lineage. Error bars in B, D, E and F show the mean ± standard deviation. Scale bars in A and C are 5 μm.

Table S1. List of statistically significant EFL-3 NanoDam peaks.

Available for download at
https://journals.biologists.com/dev/article-lookup/doi/10.1242/dev.204546#supplementary-data

Table S2. List of putative EFL-3 target genes in seam cells.

Available for download at
https://journals.biologists.com/dev/article-lookup/doi/10.1242/dev.204546#supplementary-data

Table S3. List of *C. elegans* strains, smFISH probes, oligos and vectors used in this study.

Available for download at
https://journals.biologists.com/dev/article-lookup/doi/10.1242/dev.204546#supplementary-data
